# Mitochondrial DNA and trade data support multiple origins of *Helicoverpa armigera* (Lepidoptera, Noctuidae) in Brazil

**DOI:** 10.1038/srep45302

**Published:** 2017-03-28

**Authors:** Wee Tek Tay, Thomas K. Walsh, Sharon Downes, Craig Anderson, Lars S. Jermiin, Thomas K. F. Wong, Melissa C. Piper, Ester Silva Chang, Isabella Barony Macedo, Cecilia Czepak, Gajanan T. Behere, Pierre Silvie, Miguel F. Soria, Marie Frayssinet, Karl H. J. Gordon

**Affiliations:** 1 CSIRO, Black Mountain Laboratories, Clunies Ross Street, ACT 2601, Australia; 2 CSIRO, Myall Vale Laboratories, Kamilaroi Highway, Narrabri, NSW 2390, Australia; 3 Biological and Environmental Sciences, University of Stirling, Stirling, FK9 4LA, UK; 4 Research School of Biology, Australian National University, Acton, ACT 2601, Australia; 5 Universidade de São Paulo, Instituto de Biociências, São Paulo, SP, 05508-090, Brazil; 6 Universidade Federal de Minas Gerais, Faculdade de Farmácia, Belo Horizonte, MG, 31270-901, Brazil; 7 Universidade Federal de Goiás, Escola de Agronomia, Goiânia, GO, 75804-020, Brazil; 8 Division of Crop Protection, ICAR Research Complex for North East Hill Region, Umroi Road, Umiam, Meghalaya, 793103, India; 9 IRD, UMR EGCE, FR-91198 Gif-sur-Yvette Cedex, France; 10 CIRAD, UPR AÏDA, F-34398 Montpellier Cedex 05, France; 11 Bayer S.A., Crop Science Division, São Paulo, SP, 04779-900, Brazil; 12 DGIMI, INRA, Université Montpellier, Montpellier, 34095, France

## Abstract

The Old World bollworm *Helicoverpa armigera* is now established in Brazil but efforts to identify incursion origin(s) and pathway(s) have met with limited success due to the patchiness of available data. Using international agricultural/horticultural commodity trade data and mitochondrial DNA (mtDNA) cytochrome oxidase I (COI) and cytochrome *b* (Cyt *b*) gene markers, we inferred the origins and incursion pathways into Brazil. We detected 20 mtDNA haplotypes from six Brazilian states, eight of which were new to our 97 global COI-Cyt *b* haplotype database. Direct sequence matches indicated five Brazilian haplotypes had Asian, African, and European origins. We identified 45 parsimoniously informative sites and multiple substitutions per site within the concatenated (945 bp) nucleotide dataset, implying that probabilistic phylogenetic analysis methods are needed. High diversity and signatures of uniquely shared haplotypes with diverse localities combined with the trade data suggested multiple incursions and introduction origins in Brazil. Increasing agricultural/horticultural trade activities between the Old and New Worlds represents a significant biosecurity risk factor. Identifying pest origins will enable resistance profiling that reflects countries of origin to be included when developing a resistance management strategy, while identifying incursion pathways will improve biosecurity protocols and risk analysis at biosecurity hotspots including national ports.

Global growth, food security and prosperity of agricultural communities are closely connected to international trade in agricultural and horticultural commodities. The volume and value of this trade is influenced by both climatic and pest/pathogen factors and there is interest in increasing the diversity and volume of trade whilst minimizing the impact of pests and pathogens. This conflict of interests poses a number of challenges, both before and after the incursion of a pest or pathogen into a new location.

When ascertaining the order of events that led to the incursion of a pest or pathogen, understanding the roles of propagule pressure and geographical origins is very important, especially in revealing likely incursion pathways. This, in turn, enables a more detailed assessment of factors that might have contributed to breaches in biosecurity protocols, and may assist the development of appropriate strategies for managing and reducing future incursions. Incursions of insect pests associated with international trade in agricultural and horticultural commodities are likely to increase in frequency due to globalisation and increased transport networks^1^. This highlights the importance of differentiating trade-related ‘unintended’ incursions from malicious introductions of pest species (sometimes described as agricultural bioterrorism).

Agricultural bioterrorism is defined as deliberate damage to plant crops and livestock or intentional introduction of pests or pathogens, with an aim to cause fear and negatively impact food security, human, animal and plant health, and the economy of the targeted country^2^,^3^. Agricultural bioterrorism could also involve the introduction of organisms with genes of biosecurity importance (i.e., those associated with insecticide/herbicide resistance). Developing guidelines and policies on agricultural bioterrorism is an area rapidly gaining attention due to its potential impact to global food security and socio-economic stability^4^. Accurate assessments that differentiate between natural and/or accidental introduction of pests and pathogens from that of malicious introductions will require analyses of international trade routes and activities, and a sound scientific knowledge of pests, including their invasive biology and evolutionary genetics.

The genetic diversity of a recently invaded pest species offers clues to the number of founder lineages (e.g.^5^,^6^,^7^), and incorporating information from other parts of its geographical range will allow more accurate inference of origins and incursion pathways (e.g.^6^,^8^,^9^,^10^). When a high number of individuals are introduced into a region (consecutively or concurrently; and/or over multiple occasions), the species is more likely to establish (i.e., high propagule pressure) and become invasive, for both demographic and genetic reasons^11^,^12^,^13^,^14^. The number of incursions preceding an invasion can be inferred using mitochondrial DNA (mtDNA), which allows us to estimate the number of genetically unique female founders (e.g.^5^,^6^,^8^,^15^). Usually, it is assumed that each unique mtDNA haplotype at an affected location represents a separate and independent incursion event (e.g.^16^,^17^); and assumption that seems sensible especially when the introduced populations are found in distant locations and each population has a unique mtDNA haplotype signature (e.g.^17^,^18^).

*Helicoverpa armigera* is a major polyphagous agricultural pest, with a propensity to develop insecticide resistance (e.g.^19^,^20^; see also^21^,^22^), and the ability to disperse over great distances under favourable conditions (e.g.^23^). Previously endemic to the Old World (Asia, Africa, Europe) and Australasia, it has now been reported in Argentina^24^ and Paraguay and Uruguay^25^, since its initial detection in Brazil^26^ with a likely arrival date of between 2006^27^ and 2008^28^. Long-distance dispersal may explain how ancestors of *H. zea* arrived in the New World^29^, and gene flow analyses of *H. armigera* (e.g., Israel and Turkey^30^; Europe (e.g., France, Portugal) and Africa (e.g., Tunisia, Morocco, Burkina Faso, Ivory Coast)^31^ suggest occurrence of long-distance migration between Africa, Europe and western Asia. Movements from Africa to Ascension Island (~1,600 km), and between Australia and New Zealand (∼2,200 km) are also known^32^,^33^. Genetic analysis of Brazilian *H. armigera*^27^ identified multiple female founders and indicated higher than expected genetic diversity among introduced populations, subsequently confirmed by other studies^34^,^35^. Economic losses in Brazil from this pest incursion have been estimated at US dollars (USD$) 2 billion for 2012 to 2014^36^.

Pathways of incursion by *H. armigera* into the New World are hypothesised to be associated with international agricultural trade routes^27^, however this hypothesis remained untested. In this paper, we provide a detailed analysis of mtDNA gene diversity in Brazilian *H. armigera* populations sampled during the 2012/13 early incursion period. We examined the concatenated partial mtDNA COI-Cyt *b* genes in previously studied samples from Australia, China, India, Pakistan, Uganda, Burkina Faso^29^,^37^, and include also new material from New Zealand, France, Spain, Madagascar, Ghana, Cameroon and Senegal, with the aim of consolidating relevant mtDNA data at the individual and population levels, for the purpose of identifying potential geographical origin(s) of the Brazilian incursion(s). We then consider international agricultural and horticultural trade data into Brazil to identify patterns that might enable testing of the ‘incursion pathways via international trade routes hypothesis’ and to corroborate inferred incursion pathways. These data are discussed in terms of the biological invasion processes and the potential of elucidating appropriate insecticide and Bt resistance management practices for *H. armigera* in Brazil.

## Material and Methods

### Sample collection and DNA extraction

**Figure 1:**
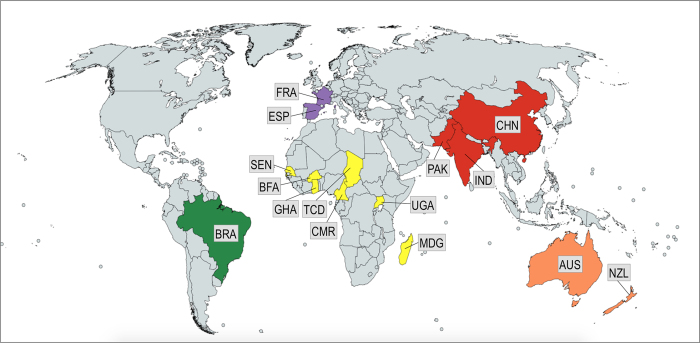
*Helicoverpa armigera* sampling sites from Brazil (BRA) (States of Goiás, Minas Gerais, Paraná, Bahia, Maranhão, Mato Grosso), Europe (France (FRA) (Montpellier, French Corsica), Spain (ESP) (Seville)), Africa (Ghana (GHA), Uganda (UGA), Burkina Faso (BFA), Chad TCD), Madagascar (MDG), Cameroon (CMR), Senegal (SEN)), Australasia (Australia (AUS) (State of Victoria), New Zealand (NZL) (Auckland)), Asia (China (CHN) (Shandong Province), India (IND) (States of Punjab, Maharashtra, Andhra Pradesh, Tamil Nadu, Telangana), and Pakistan (PAK) (Multan). Map created using Mapchart https://mapchart.net. See Supplementary Table 1 for sampling details].

*Helicoverpa armigera* samples were collected either as larvae (from host plants) or adults (by light/pheromone traps) from Asia, Australasia, Africa, and Europe (n = 329; collected between 2001–2014), and from Brazil (n = 114; collected between March and August, 2013). Samples were preserved in ethanol (>95%) prior to DNA extraction using the Qiagen Blood and Tissue Kit. Taxonomic identity of each specimen was confirmed using molecular DNA markers i.e., using the mtDNA COI and Cyt b genes, as per^37^ (Fig. 1; Suppl. Table 1).

### Molecular characterisation of the samples

PCR amplification of partial mtDNA COI and Cyt *b* genes was done as reported^27^,^37^. Amplicons were confirmed in 1.5% agarose gels containing 1% w/v GelRed prior to Sanger sequencing (Macrogen Inc., Seoul, Rep. of Korea; Biological Resources Facility, Australian National University, Canberra, Australia). DNA trace sequences were assembled using Staden Pregap 4 and Gap 4^38^. Species identity was confirmed through BLASTN searches against the non-redundant DNA database in GenBank^39^. Estimates of haplotype (*h*) and nucleotide (*π*) diversity for Brazil, Old World, and at the global level were inferred using DnaSP^40^. A haplotype network for the 97 unique mtDNA haplotypes identified from this study was inferred using TCS^41^ within PopART^42^.

### Molecular data survey and phylogenetic analysis

Partial COI and Cyt *b* gene sequences were aligned prior to concatenation to form a master alignment (443 sequences; 945 base pairs (bp)), and surveyed for evidence of multiple substitutions at the same sites to minimise biases to phylogenetic estimates. Evidence of multiple substitutions was analysed using Reticulate^43^ to yield a compatibility plot (Suppl. Fig. 3), and the program dnapars^44^ was used to infer the most parsimonious number of substitutions that might have occurred at the parsimony-informative sites (Suppl. Fig. 4). Analysis was repeated 10,000 times to increase the chance of finding the most parsimonious estimate.

Phylogenetic analysis based on 134 aligned sequences (after removal of surplus identical sequences from the master alignment) involved a probabilistic maximum-likelihood approach to enable handling of detected multiple substitutions. ModelFinder^45^ and the AICc optimality criterion were used to find the optimal sequence evolution model. IQ-TREE^46^ was used to infer the most likely phylogeny with 10,000 replications, and the consistency of the phylogeny estimated using ultrafast bootstrap^47^ with default settings. This enabled assessment of whether different sets of sites, drawn at random with replacement from the sub-alignment, generated a consistent phylogenetic result.

As geographical locations were unevenly sampled (i.e., ranging from 3 to 58 sequences/location), the potential impact of this uneven sampling effort on the phylogeny was evaluated using jackknifing. First, 200,000 sub-alignments that consisted of three sequences from each location were generated randomly from the master alignment using AliJack http://github.com/thomaskf/AliJack to produce sub-alignments with 104 sequences. Next, the most likely trees from these sub-alignments were inferred using IQ-TREE and the HKY + R2 model. Finally, from the inferred trees we computed the ratio, *J*, between (i) the number of trees with selected sequences forming a clade, and (ii) the number of trees with the selected sequences included. The jackknife score (i.e., product 100 × *J*) was obtained using CheckJack http://github.com/thomaskf/CheckJack.

### Trade data

Trade data (value in USD$), were obtained from the Observatory of Economic Complexity (OEC) website^48^. The OEC compiled its source data from the United Nations Statistical Division (COMTRADE) for the period 2001–2013 using repository of official trade statistics, and as compiled by the International Merchandise Trade Statistics Section of COMTRADE. 2002–2013 trade data were extracted for live horticultural and agricultural commodities imported from the Old World to Brazil, to quantify the movement of potentially contaminated plant material and agricultural products. Trade data classified as ‘Live Trees and Other Plants’ (Harmonized System [HS] code 06; Suppl. Table 3), ‘Edible Vegetables and certain roots and tubers’ (HS code 07; Suppl. Table 4), and ‘Edible Fruits and Nuts, Peel of Citrus/Melons’ (HS code 08; Suppl. Table 5) were used. Countries were grouped into respective continents (Africa, Asia, Australasia, Europe, North America, South America) as per the OEC website (see Suppl. Tables 3, 4 and 5). Data excluded import activities from North and South American countries into Brazil (see Suppl. Tables 3, 4 and 5) due to the presumed pre-2006 absence of *H. armigera* in these regions.

### Import risk factor analysis

We assessed the biosecurity import risks of *H. armigera* with respect to trade data, the import risk/likelihood of entry, population establishment and its subsequent spread against suitable commodities (i.e., HS codes 06, 07, 08) using Biosecurity Australia’s import risk analyses method^49^. This method considered trade volume (as measured by value) in a year to estimate pest entry likelihood^49^. Based on eight specific criteria (i.e., (*i*) ecological specificity, (*ii*) plant host availability and suitability, (*iii*) survey methodology, (*iv*) taxonomic recognition, (*v*) entry potential, (*vi*) destination of infested material, (*vii*) potential economic impact, and (*viii*) establishment potential), *H. armigera* has been rated as a ‘High’ risk pest to the New World^50^. We assessed *H. armigera*’s import risk on a per-year basis for the period 2002–2013. As the overall trade volumes are expected to grow over time, this in turn would lead to the increase in the likelihood for both pest entry and population establishment^49^. Equation (1) (see from^49^) was applied with time (*t*) replaced by volume (*ϑ*), to estimate the likelihood of incursion (‘incursion’ as per ‘incursion’ as per^49^) and establishment by *H. armigera*:


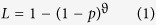


where *L* is the likelihood of incursion, *p* is the probability of incursion when importing one unit of volume of the product^51^, and *ϑ* is product units (note that 1−*p* represents the probability that *H. armigera* will not invade a location in a particular year of trade).

We inferred the volume units by calculating the ratio per commodity (i.e., HS codes 06, 07, 08) and for overall trade volume (HS codes 06 + 07 + 08), by dividing individual trade volumes per region (i.e., Asia, Africa, Europe, Australasia) per year by the highest trade volume for the HS code commodity in question (i.e., such that the highest volume for the particular HS code is equal to 1). This assessment of likelihood based on a semi-quantitative method relies on language-based likelihoods by intervals of probability (i.e., ‘high’ = 0.7–1.0; ‘Moderate’ = 0.3–0.7; ‘Low’ = 0.05–0.3)^49^,^51^. For *H. armigera*, we used the probability threshold of 0.7 to conservative represent the ‘lower end’ of high-risk likelihood of an incursion.

## Results

### MtDNA diversity

We analysed 945 bp (i.e., 511 bp COI; 434 bp Cyt *b*) from 443*H. armigera* individuals (Brazil: *N* = 114; Old world/Australasia: *N* = 329) and identified 97 haplotypes, including 20 from Brazil (Suppl. Table 1). These samples were compared to those reported previously^27^,^29^, with additional samples from Chad (*N* = 6), Cameroon (*N* = 13), Senegal (*N* = 11), Ghana (*N* = 4), Madagascar (*N* = 14), Spain (*N* = 6), France/French Corsica (*N* = 17), and New Zealand (*N* = 9) (Suppl. Table 1) also included in our analyses. 21 new mtDNA COI (GenBank: KX494879-KX494899) and 10 new Cyt *b* haplotypes (GenBank: KX494900-KX494909) were identified in Senegal, Madagascar, Chad, Cameroon, Spain, French Corsica, New Zealand, and Brazil (Suppl. Table 2). Concatenating the partial gene haplotypes generated the 97*H. armigera* haplotypes mentioned above, and eight of the 20 Brazilian haplotypes have never been reported (Suppl. Table 2).

### Mitochondrial DNA haplotype diversity of *H. armigera*

Estimates of haplotype and nucleotide diversity among samples of *H. armigera* from Brazil, the Old World, and the globe are provided in Table 1. The haplotype diversity estimate for Brazil was 0.753, while that for the Old World was 0.820. However, these values overlap when their standard deviations are taken into account. The haplotype network showed a high level of complexity, but lacked obvious population/haplotype cluster patterns (Suppl. Fig. 1). Five haplotypes were found in Brazil and one other country (i.e., Madagascar, Chad, India, China, France), implying five possible sources for incursions into Brazil. The lack of distinct clusters within Brazil is consistent with earlier studies (e.g.^27^,^29^,^35^,^36^) involving the new Brazilian populations and indicating high mobility in this species.

Trade data on horticultural and agricultural commodities into Brazil showed that we lacked samples from countries with significant export or re-export activities to Brazil (e.g., Israel, Tunisia, Turkey, Thailand, Egypt, South Africa, Nigeria, Italy, The Netherlands, Greece), with these countries being possible origins of novel and unique Brazilian haplotypes. Our mtDNA analysis also identified globally distributed haplotypes (e.g., Hap0101, Hap0108, Hap0111, Hap0201; Suppl. Tables 1 and 2; Suppl. Figs 1 and 2), suggesting that while some haplotypes might allow us to identify origins of introduction, others are too widespread to offer an insight into incursion sources.

### Phylogenetic analysis of the molecular data

**Figure 2:**
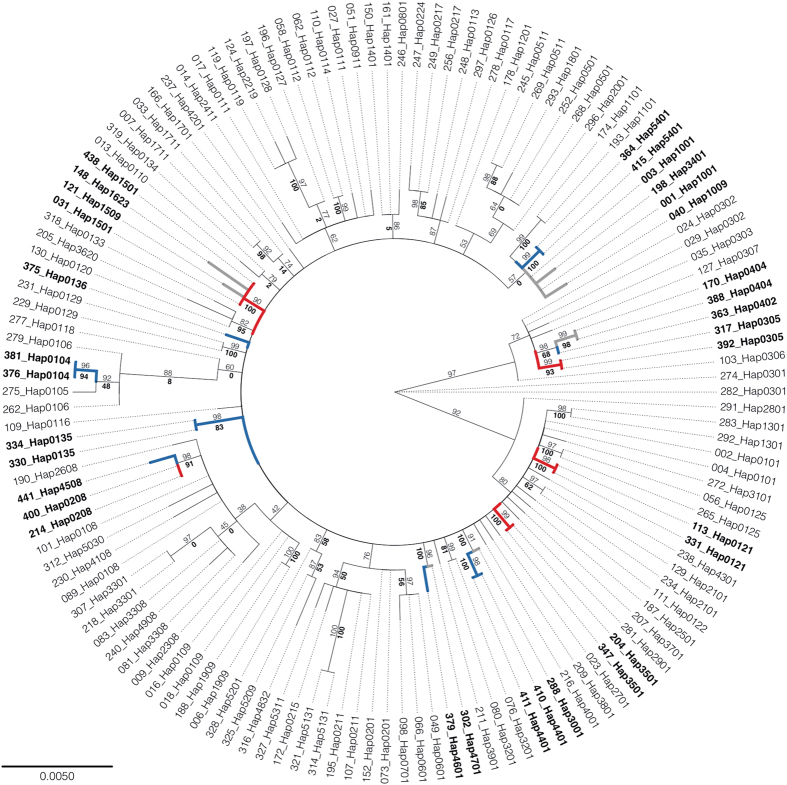
Phylogenetic relationships among the 97 *Helicoverpa armigera* mtDNA haplotypes (some haplotypes are in duplicate to ensure that parsimony-informative splits in the data are represented in the tree). Names of sequences are represented by individual ID (see Suppl. Table 1) followed by haplotype ID (see Suppl. Table 2). Sequence discussed in the main text are in bold. Blue edges indicate Brazilian unique haplotypes; red edges indicate haplotypes found in Brazil and one other country; grey edges indicate haplotypes found in Brazil and more than one other country. Bootstrap scores (from 10,000 replicates) for selected edges are shown in a light font; jackknife scores (from 200,000 replicates) for selected edges are shown in a bold font.

The inferred phylogeny (Fig. 2; HKY + R2 optimal sequence evolution model) is characterized by an abundance of polytomies, likely due to the low number of parsimony-informative sites. In some cases, the bootstrap scores (indicated for selected edges) are high (e.g., 321_Hap5131; 314_Hap5131), implying that the data are phylogenetically consistent for these sequences, but in the majority of cases are far below 100% due to phylogenetically inconsistent sequence data. The jackknife scores (i.e., for assessing the impact of incomplete taxon sampling) were generally high (Fig. 2), suggesting that our taxon sampling for this study is unlikely to be an issue.

The phylogeny reveals that the eight unique Brazilian haplotypes are often not as closely related to one another as they are to a haplotype found in Australia (Hap1001, Hap3001, Hap0404 or Hap0105), New Zealand (Hap4701), China (Hap1001), India (Hap1001 or Hap1009), Burkina Faso (Hap1001), Uganda (Hap0404) or Senegal (Hap3401). Of these seven non-Brazilian haplotypes (i.e., Hap1001, Hap3001, Hap0404, Hap0105, Hap4701, Hap1009, Hap3401) found in distant locations, five are reported from only one country (i.e., Australia, New Zealand, India, Senegal). Therefore, it is possible that these eight uniquely Brazilian haplotypes could have originated from these populations, and perhaps also populations in Uganda, Burkina Faso, and China (Suppl. Table 1), as supported also by genome-wide SNP data and pyrethroid resistance gene analysis^52^.

The phylogeny also reveals five haplotypes found in Brazil and one other country (i.e., France, Senegal, Ghana, India, and China). This result again suggests separate incursion events in addition to those mentioned above. Similarly, seven haplotypes were found in Brazil and in more than one other country (Suppl. Table 2). The geographical distributions of these seven haplotypes also hint of multiple incursions (i.e., ≥three countries), however, the evolutionary relationship among these seven haplotypes is unclear (Fig. 2). Taken as a whole, mtDNA haplotype analyses support multiple and perhaps continuous incursions of *H. armigera* into Brazil. At this early incursion stage, there exist at least 20*H. armigera* maternal lineages in Brazil. Of these, eight are unique to Brazil and their close evolutionary relationship with haplotypes found in other countries suggests that they are unlikely to be the result of recent divergence (*c.f*.^35^).

### Trade data

Trade data from 2002–2013 indicate an approximately fourfold increase in activity from Asia into Brazil with limited imports originating from Australasia (Australia, New Zealand). Within Asia; India, Thailand, Israel, Turkey and China were the main trading partners although others (e.g., Japan, Malaysia, South Korea, Sri Lanka, Lebanon, Saudi Arabia, United Arab Emirates, Syria) also had significant trade activity with Brazil over the surveyed period.

Significant exports to Brazil of HS code 06 commodities (Living Trees and Other Plants; Suppl. Table 3) were identified from European countries (Suppl. Fig. 5), including The Netherlands, France, Italy, Spain, Germany, Belgium, Luxemburg, Denmark, Greece, Portugal, Great Britain, and Ireland. During the 12-year period surveyed, the African continent exported high volume of HS code 07 commodities (Edible Vegetables, including Certain Roots and Tubers; Suppl. Table 4). Over the period 2007–2012, there were significant imports of fresh or chilled vegetables from Egypt (Suppl. Fig. 6), with a drop in (or absence of) this trade from Europe and Asia. European countries exported high volumes of HS code 08 commodities (Edible Fruits and Nuts, Peel of Citrus/Melons; Suppl. Table 5) to Brazil, with a fourfold increase in trade since 2002 from Asia and a sevenfold increase in trade volume from New Zealand (Suppl. Fig. 7).

### Trade volume and associated biosecurity import risk analysis

**Figure 3:**
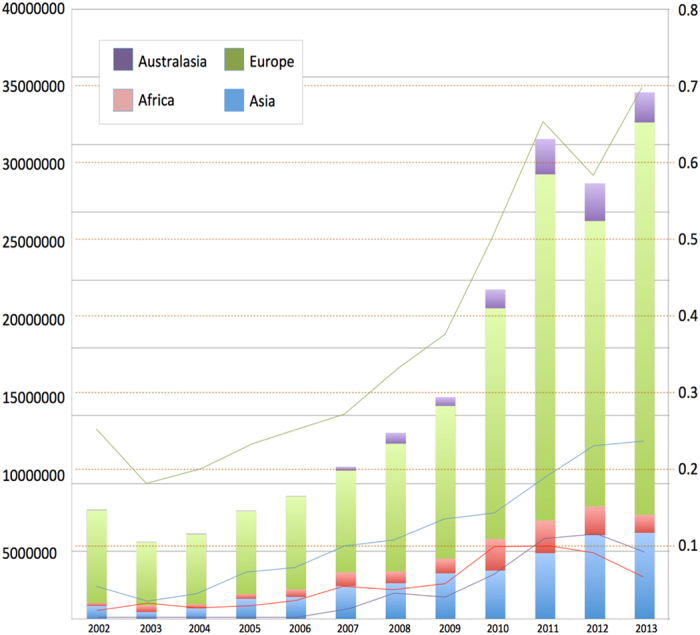
Combined export trade volume from Asia, Africa, Europe and Australasia continents to Brazil. Data are for 12-years period (2002–2013; X-axis) for Harmonized commodity description and coding system (HS) codes 06, 07 and 08 (Suppl. Tables 3, 4 and 5) in USD$ (left Y-axis). Biosecurity entry risk factor (orange doted lines, right Y-axis) for *H. armigera* that considered introduction, establishment and population spread as calculated for trade volume per year by continental regions are also shown.

The biosecurity import risks of *H. armigera* increased expectedly with increasing trade volume (Fig. 3; Suppl. Figs 5, 6 and 7). For HS code 6 commodities, there was a moderate-to-high import risk (i.e., likelihood from 0.42 to 0.70) between 2008 and 2012 for products from Europe, followed by products from Asia with a likelihood of import risk that rapidly increased from ‘low’ (i.e., 0.07) in 2008 to moderate (i.e., 0.37) in 2012. For HS code 07 commodities, the likelihood of the *H. armigera* import risk from African products spiked at 0.7 (i.e., high) in 2010, and at ‘moderate risk levels’ (i.e., 0.3, 0.4 and 0.5) in 2002, 2007, and 2012, respectively, from Asian products. Import risks from *H. armigera* for HS code 08 commodities was the greatest from Europe, rapidly increasing from 0.15 (i.e., ‘low’) to 0.7 (i.e., ‘high’) over the 12 years surveyed, while those for the other three continents also grew from ≤0.01 (i.e., negligible/very low) to approximately 0.16 (i.e., low). When combining all three HS codes of commodities (Fig. 3), the biosecurity import risk likelihood was similar to that for HS code 08, although the likelihood was slightly higher (but still ‘low’) for Asia (0.03 in 2002, to 0.23 in 2012), Australasia (0.0003 in 2002, to 0.09 in 2012) and Africa (0.008 in 2002, to 0.05 in 2012).

## Discussion

By combining multiple mtDNA markers and increasing global sampling sites, we identified Asia (i.e., China, India), Africa (i.e., Madagascar, Senegal) and Europe (i.e., France) as potential origins of Brazilian *H. armigera*, and inferred incursion pathways by combining genetic and trade data. This approach may be relevant to other invasive organisms with high effective population sizes, such as the soybean stem fly (SSF) *Melanagromyza sojae*^53^, the whitefly *Bemisia tabaci* cryptic species complex (e.g.^54^), the spotted-wing vinegar fruit fly *Drosophila suzukii*^55^, the European Grapevine moth *Lobesia botrana*^56^,^57^, and the Russian Wheat Aphid *Diuraphis noxia* and incorporating genetic signatures of endosymbiont bacteria (e.g.^58^), amongst other.

Leite *et al*.^35^ compared the standard barcode mtDNA COI region of Brazilian *H. armigera* to publicly available mtDNA COI haplotypes from the Old World, and identified Asian (i.e., China) and European maternal lineages in Brazil, based on shared haplotypes. Africa, Australia and Pakistan were not identified as potential origins due to poor availability of sequence data from these countries. Furthermore, common global haplotypes shared widely between Brazil and Old World locations also reduced the power of detecting potential origins. We showed that these regions, especially China, India, and France, were significant export countries of live host material capable of supporting *H. armigera* into Brazil, thereby supporting the notion of increased international trade activities as risks factors^1^,^59^.

Rumours of ‘agricultural bioterrorism’ have surfaced periodically in Brazil (e.g., Jornal diz que ocorrência de lagarta pode ser bioterrorismo; New threat to Brazil’s breadbasket: a pesky caterpillar; The Helicoverpa armigera). The diverse origins of *H. armigera* detected in Brazil would mean it is highly unlikely there can have been a deliberate release of this pest. Previously, Spanish plant products were identified as the commodity category where most *H. armigera* interceptions occurred in the EU^60^. This is in general agreement with our trade data patterns, although we also identified France, Italy and The Netherlands as countries with significant export activities to Brazil. Since 2008*H. armigera* has been ‘deregulated’ in Europe for trade involving cut flowers from Africa^61^. Much of these horticultural/agricultural goods are exported to third countries including Brazil. This suggests that Europe might also be the route of introduction, and highlights the potential global impact of regional phytosanitary policies. The general lack of agricultural trade with Australia could explain the absence of unique Australian haplotypes in our Brazilian data. Interestingly, *H. armigera* was detected in 2014 in *Capsicum* sp. from the Dominican Republic into the EU^62^, a first reported incidence of a New World to Old World reintroduction.

A common finding between previous (e.g.^27^,^34^,^35^) and this study is the high genetic mtDNA haplotype diversity in the Brazilian populations, which is unusual for a recently introduced pest. Eight haplotypes (Suppl. Tables 1 and 2) represented unique maternal lineages not currently known from Old World populations. Matching of these to better identify additional potential origins of *H. armigera* in Brazil will require sampling countries with substantial agricultural commodity exports to Brazil. Despite the lack of substantial trade in agricultural and horticultural commodities with Brazil, a Malagasy haplotype (Hap0208) was identified. Population movement of *H. armigera* between Madagascar and neighbouring African nations is currently unknown but significant trade does occur between South Africa and Brazil. It may be that this haplotype represents a general ‘southern African’ haplotype and is present in both South Africa and Madagascar.

The discovery of Asian and African/European haplotypes offers unique insights into two potential introduction pathways in Brazil: natural (chance) dispersals, and human-mediated dispersal. Our finding that Senegal, Madagascar, French Corsica and Brazilian populations share the same unique haplotypes could indicate chance dispersal from the African continent, as well as human mediated introductions from Asia and Europe. Insect dispersals from Africa to the New World are hypothesised to associate with easterly winds (e.g.^63^). Knowing the potential origins of *H. armigera* in Brazil will provide valuable insights for its managing in the New World. In Cameroon, China and India, *H. armigera* carries resistance to synthetic pyrethroids^64^,^65^,^66^, organophosphates^67^,^68^ and carbamates^69^,^70^. Resistance to diverse classes of pyrethroids has also been reported in populations from Benin (west Africa)^71^. In southern France, pyrethroid-resistance *H. armigera* was also reported^72^,^73^, while populations resistant to pyrethroids, organophosphates and carbamates were identified in Spain^74^,^75^. Significant reduced susceptibility to Cry1Ac has also been detected in some Chinese *H. armigera* populations^76^, and *H. armigera* larvae on Bt cotton (BG-II) which has *cry1Ac*+ *cry2Ab* genes exists in India^77^.

In Brazil, farmers reported difficulties managing *H. armigera* and overused insecticides to restrict its outbreak^78^, potentially reflecting insecticide resistances in the introduced populations. This is supported by Anderson *et al*.^52^ where Brazilian *H. armigera* grouped with Asian individuals for an allele for pyrethroid resistance in *H. armigera* sampled during this early stage of incursion. Population-wide studies (e.g.^52^) will therefore provide a starting point for prioritising screens for resistance in chemistries (organophosphates, carbamates, Cry1Ac, Cry2Ab) that are likely to be present in source populations and which have high use in the New World. This information will be critical for developing strategies to manage the further evolution of insecticide resistance and for the management of *H. armigera* elsewhere, including the re-introduction of *H. armigera* back into the Old World with heightened resistance acquired in the New World, and the potential spread into the North American final frontier.

While it may have been possible to eliminate the initial *H. armigera* population(s) in Brazil (assuming single incursion event), this is now unlikely given shared geographical boundaries and similar economic activities. *H. armigera*’s presence in the New World may also have been as early as the mid-2000’s, with individuals intercepted in 2006 in a Peruvian “mange-tout” bean consignment found to possess COI haplotypes (630 bp) that matched those from China, Spain, Uganda and Egypt^79^. This suggests that incursions into Brazil may well have involved neighbouring countries, and reinforcing the need to coordinate and co-operate in the area of biosecurity. Indeed, the study of Lopes-da-Silva *et al*.^36^ has shown that at least in Brazil, there has been an increase in frequencies of incursions by exotic agricultural insect pests since the early 1900’s, with at least two more exotic insect pest incursions being reported in recent times (e.g.^80^,^81^). On-going monitoring of New World *H. armigera* populations though genetic analysis of quarantine-intercepted material will contribute to mapping its New World spread, thereby enabling early intervention via regional-specific pest management strategies to achieve their intended efficacies.

## Additional Information

**How to cite this article:** Tay, W. T. *et al*. Mitochondrial DNA and trade data support multiple origins of *Helicoverpa armigera* (Lepidoptera, Noctuidae) in Brazil. *Sci. Rep.* **7**, 45302; doi: 10.1038/srep45302 (2017).

**Publisher's note:** Springer Nature remains neutral with regard to jurisdictional claims in published maps and institutional affiliations.

## Supplementary Material

Supplementary Figures and Tables

## Figures and Tables

**Table 1:** Haplotype (*h*) and nucleotide (*π*) diversity estimates among mtDNA samples of *Helicoverpa armigera* from Brazil, the Old World, and the globe (the global data combined all populations from the Old World and Brazil), SD = standard deviation.

	No. haplotypes	*h* (±SD)	π (±SD)
Old World	75	0.820 (±0.0005)	0.0029 (±0.0003)
Brazil	20	0.753 (±0.0360)	0.0028 (±0.0005)
Global	97	0.806 (±0.0190)	0.0029 (±0.0002)
