# Supplementary material for: Mitochondrial DNA and trade data support multiple origins of Helicoverpa armigera (Lepidoptera, Noctuidae) in Brazil
Source: Sci Rep. 2017 Mar 28;7:45302. doi: 10.1038/srep45302 (PMC5368605; doi:10.1038/srep45302)
Supplement: Supplementary Figures and Tables [file srep45302-s1.pdf]

Mitochondrial DNA and trade data support multiple origins of *Helicoverpa armigera* (Lepidoptera, Noctuidae) in Brazil

Wee Tek Tay[1], Thomas K. Walsh[1], Sharon Downes[2], Craig Anderson[1,3], Lars S. Jermiin[1,4], Thomas K. F. Wong[1,4], Melissa C. Piper[1], Ester Silva Chang[1,5], Isabella Barony Macedo[1,6], Cecilia Czepak[7], Gajanan T. Behere[8], Pierre Silvie[9, 10], Miguel F. Soria[11], Marie Frayssinet[12], Karl H. J. Gordon[1]

[1] CSIRO, Black Mountain Laboratories, Clunies Ross Street, ACT 2601, Australia.

[2] CSIRO, Myall Vale Laboratories, Kamilaroi Highway, Narrabri, NSW 2390, Australia.

[3] Biological and Environmental Sciences, University of Stirling, Stirling, FK9 4LA, UK.

[4] Research School of Biology, Australian National University, Acton, ACT 2601, Australia.

[5] Universidade de São Paulo, Instituto de Biociências, São Paulo, SP, 05508-090, Brazil.

[6] Universidade Federal de Minas Gerais, Faculdade de Farmácia, Belo Horizonte, MG, 31270-901, Brazil.

[7] Universidade Federal de Goiás, Escola de Agronomia, Goiânia, GO, 75804-020, Brazil.

[8] Division of Crop Protection, ICAR Research Complex for North East Hill Region, Umroi Road, Umiam, Meghalaya-793103, India.

[9] IRD, UMR EGCE, FR-91198 Gif-sur-Yvette Cedex, France.

[10] CIRAD, UPR AÏDA, F-34398 Montpellier Cedex 05, France.

[11] Bayer S.A., Crop Science Division, São Paulo, SP, 04779-900, Brazil.

[12] DGIMI, INRA, Université Montpellier, Montpellier, 34095, France.

Correspondence

Tel: +61-2-6246 4286 | **Fax:** +61-2-6246 4000 | **Email:** weetek.tay@csiro.au

Suppl. Fig. 1: Global *Helicoverpa armigera* mitochondrial DNA (mtDNA) haplotype network inferred from concatenation of 511bp mtDNA COI partial gene and 434bp mtDNA Cyt *b* partial gene. Shared unique mtDNA haplotypes between Brazil and Madagascar, Senegal, China, India and French Corsica are indicated by red arrows. Number of base changes between haplotypes are indicated by black bars.

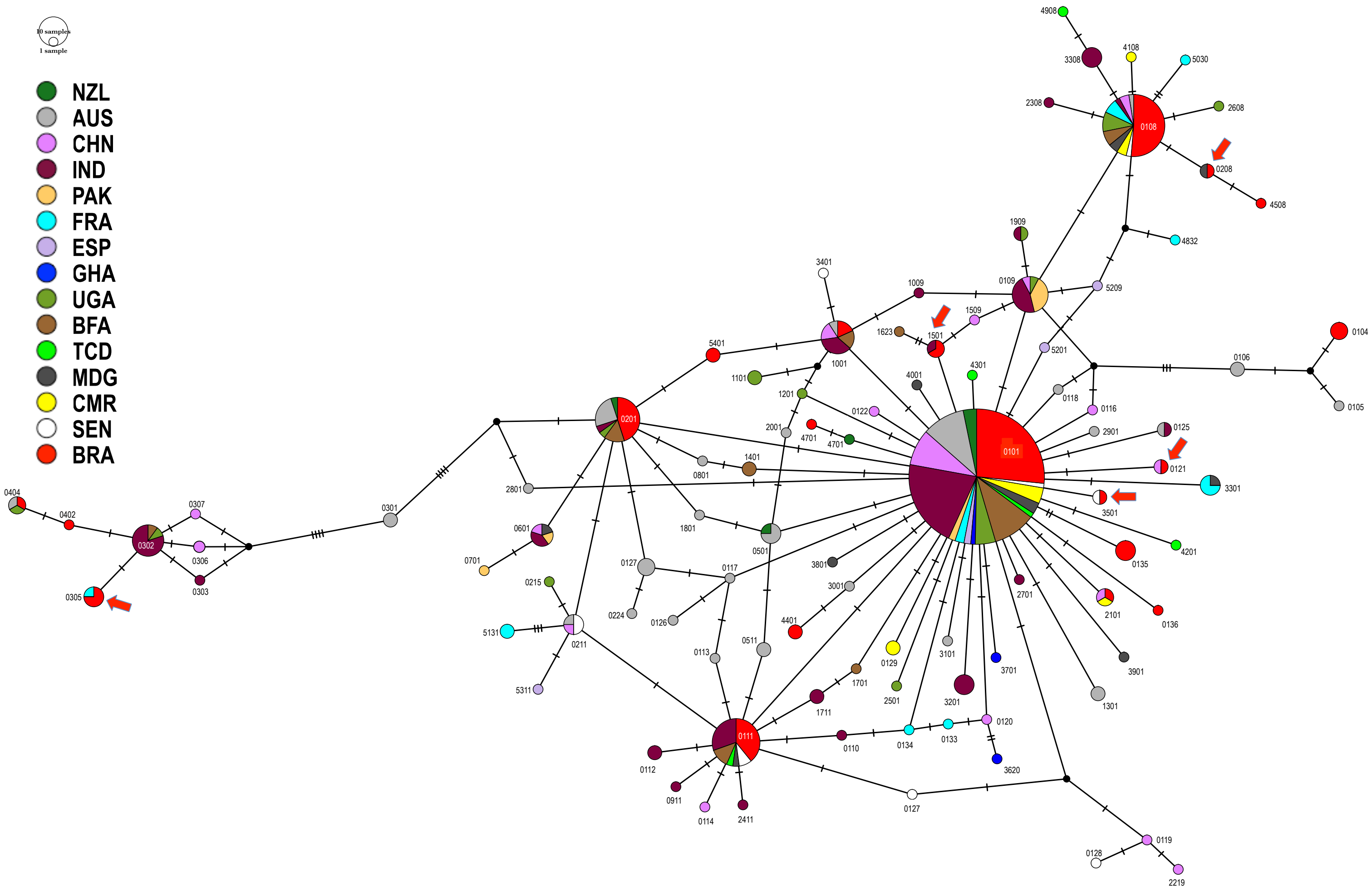

Suppl. Fig. 2: Haplotype distribution (from concatenated partial mtDNA COI-Cyt *b* genes) from sampling countries.

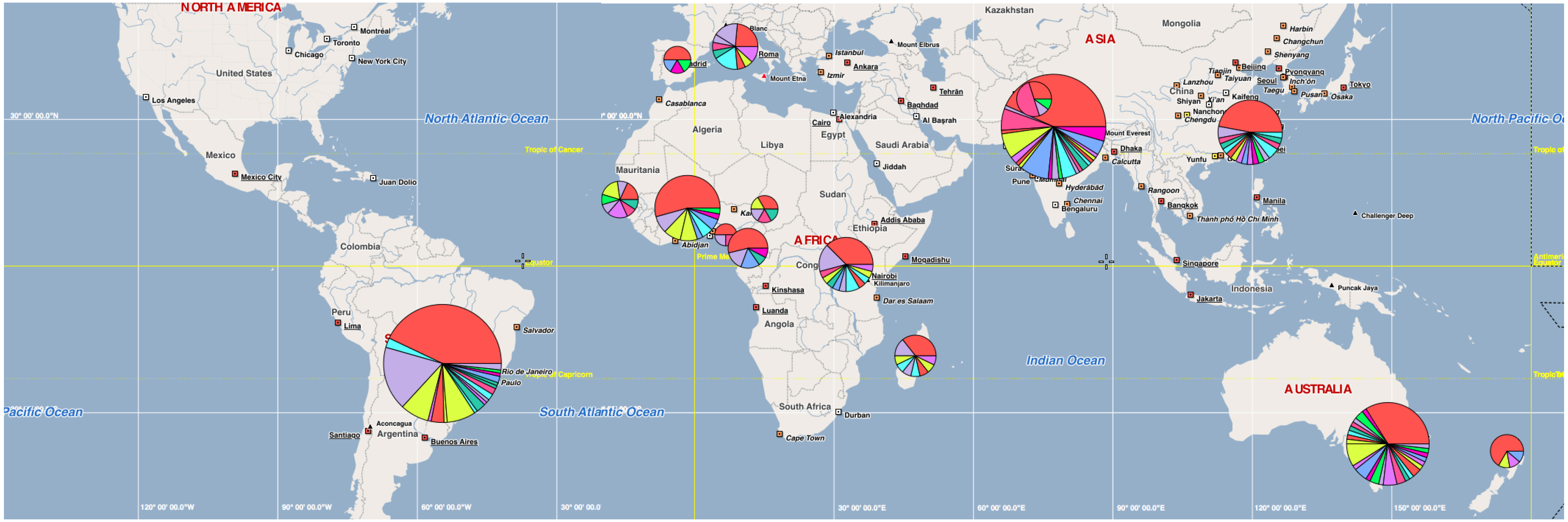

Note: The haplotype distribution map was created using the Allan Wilson Centre Imaging Evolution Initiative free open source population genetic software PopART <http://popart.otago.ac.nz/index.shtml>, superimposed on the Marble Virtual Globe (map theme: plain) using Marble Library version 0.17.0 <http://edu.kde.org> (Marble licensed under the terms of GNU LGPL version 2.1).

Suppl. Fig. 3: A compatibility plot from the master alignment was generated using the program Reticulate (see main text) to determine whether multiple substitutions have occurred at the same sites. Reticulate detects whether pairs of parsimony-informative sites are compatible or incompatible (sites are compatible if the most parsimonious distributions of their nucleotides fit the same tree). When sites are incompatible, additional substitutions will be required to fit their nucleotides to the same tree, implying that multiple substitutions must have occurred at one or both of the sites. Of the 45 parsimony-informative sites from the aligned sequences, 29 were incompatible with at least one other such site, thereby providing evidence for multiple substitutions at some sites. Compatible and incompatible parsimony-informative sites are showed by white pixels and black pixels, respectively. When two sites have evolved on the same tree and they are incompatible, then one or both of the sites must have changed at least twice since the sequences diverged from their last common ancestor.

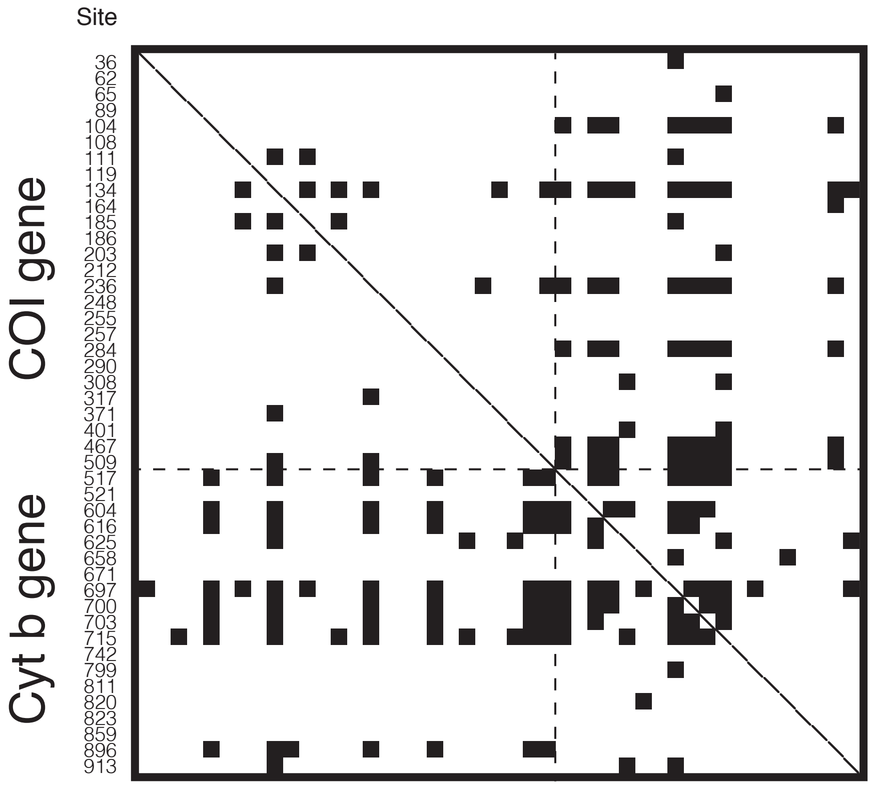

Suppl. Fig. 4: Results of how often the 45 parsimony-informative sites might have changed, with analyses identifying 47% of the sites have changed at least twice and that a small number of sites have changed at least six times since the sequences diverged from their last common ancestor. Distribution of the number of sites that have changed between one to six times since the 97 mtDNA COI-Cyt *b* haplotypes diverged from their most recent common ancestor (result is conditional on the most parsimonious tree for these data) are shown.

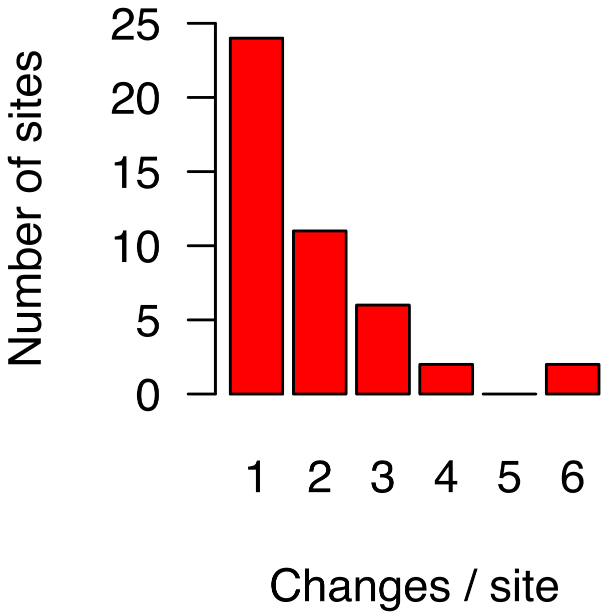

Suppl. Fig. 5: Trade volume between exporting countries from Asia, Africa, Europe and Australasia continents and Brazil. Data are for 12-years period (2002-2013; X-axis) for 'Live trees, plants, bulbs, roots, cut flowers, etc.' (Harmonized commodity description and coding system (HS code): 0602 (other live plants), 060210 (cuttings and slips, not rooted), 060310 (cut flowers, fresh), 060491 (foliage, branches for bouquets, fresh) in USD$ (left Y-axis). Biosecurity entry risk factor (right Y-axis) for *H. armigera* that considered introduction, establishment and population spread as calculated for trade volume per year by region (i.e., Europe, Africa, Asia, Australasia) are also shown. For details see Suppl. Table 3.

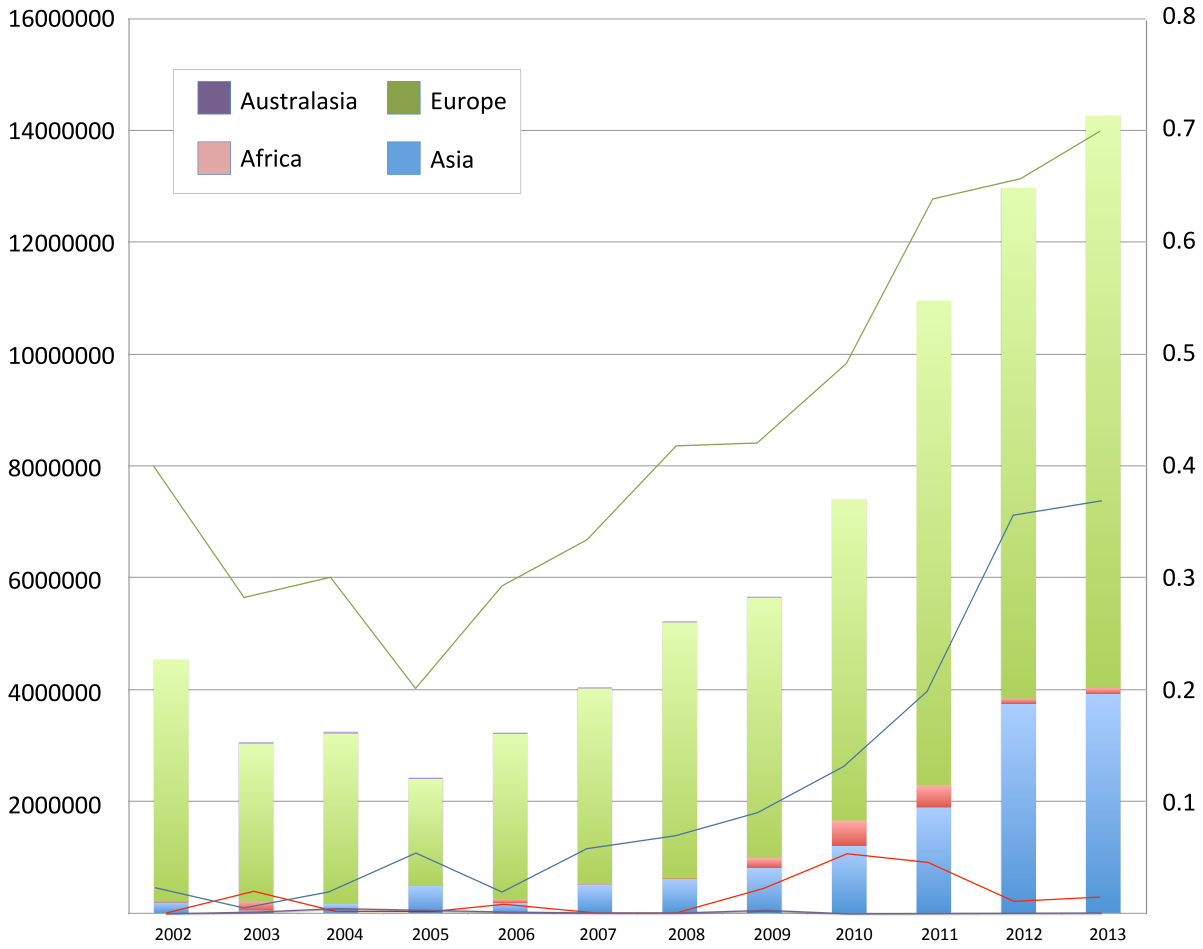

Suppl. Fig. 6: Trade volume between exporting countries from Asia, Africa, Europe and Australasia continents and Brazil. Data are for 12-years period (2002-2013; X-axis) for 'Edible vegetables and certain roots and tubers.' (Harmonized commodity description and coding system (HS code): 070390 (Leeks and other alliaceous vegetables, fresh or chilled), 070519 (Lettuce, fresh or chilled except cabbage lettuce), 070810 (peas, shelled or unshelled, fresh or chilled), 070960 (peppers (Capsicum, Pimenta) fresh or chilled), 070970 (plants, live (including their roots), not elsewhere specified (nes)), 070990 (vegetables, fresh or chilled nes) in USD$ (left Y-axis). Biosecurity entry risk factor (right Y-axis) for *H. armigera* that considered introduction, establishment and population spread as calculated for trade volume per year by region (i.e., Europe, Africa, Asia, Australasia) are also shown. For details see Suppl. Table 4.

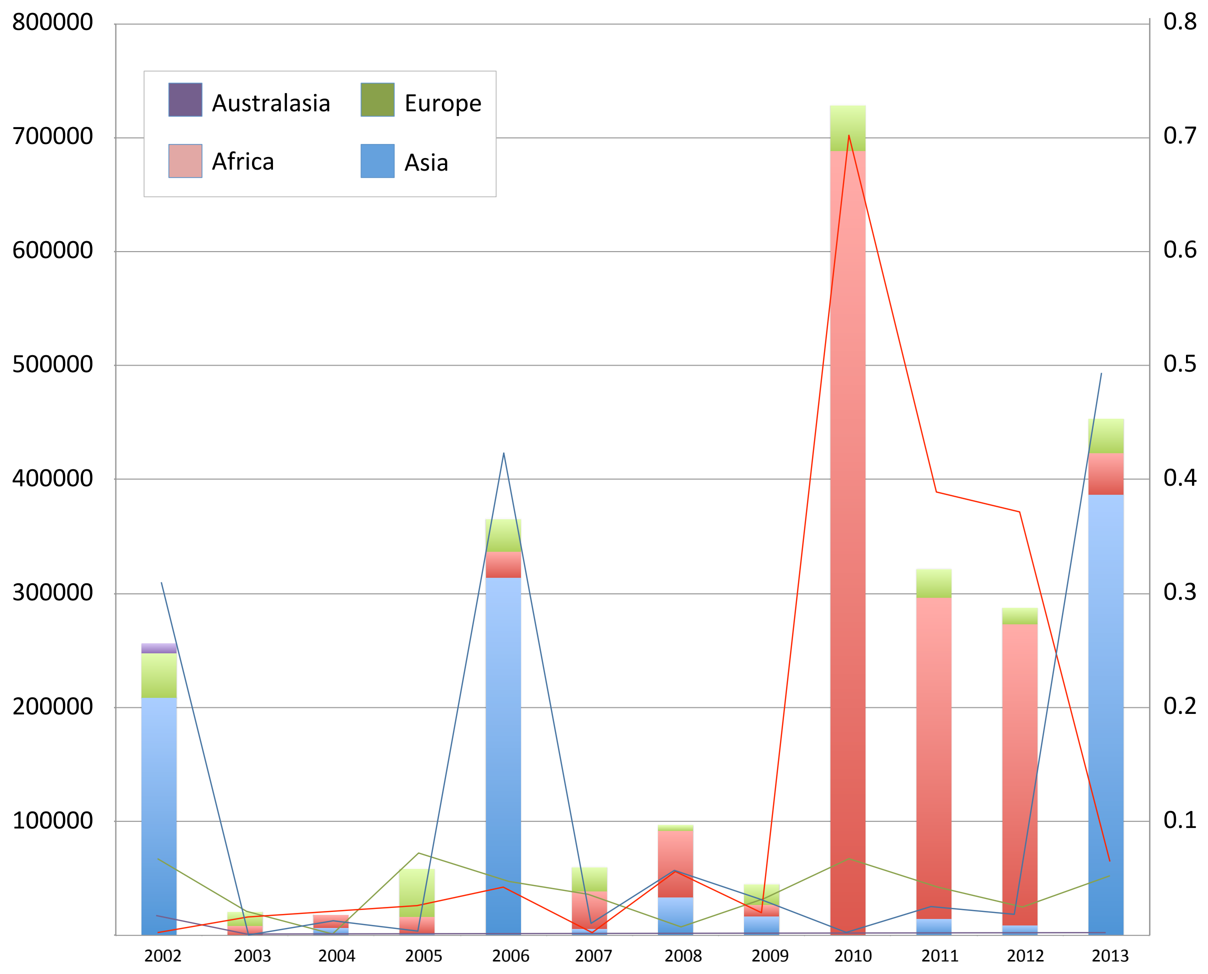

Suppl. Fig. 7: Trade volume between exporting countries from Asia, Africa, Europe and Australasia continents and Brazil. Data are for 12-years period (2002-2013; X-axis) for 'Edible fruit, nuts, peel of citrus fruit, melons.' (Harmonized commodity description and coding system (HS code): 0804 (tropical fruits), 081010 (strawberries, fresh), 081090 (fruits, fresh nes)) in units of USD$ (left Y-axis). Biosecurity entry risk factor (orange doted lines, right Y-axis) for *H. armigera* that considered introduction, establishment and population spread as calculated for trade volume per year by region (i.e., Europe, Africa, Asia, Australasia) are also shown. For details see Suppl. Table 5.

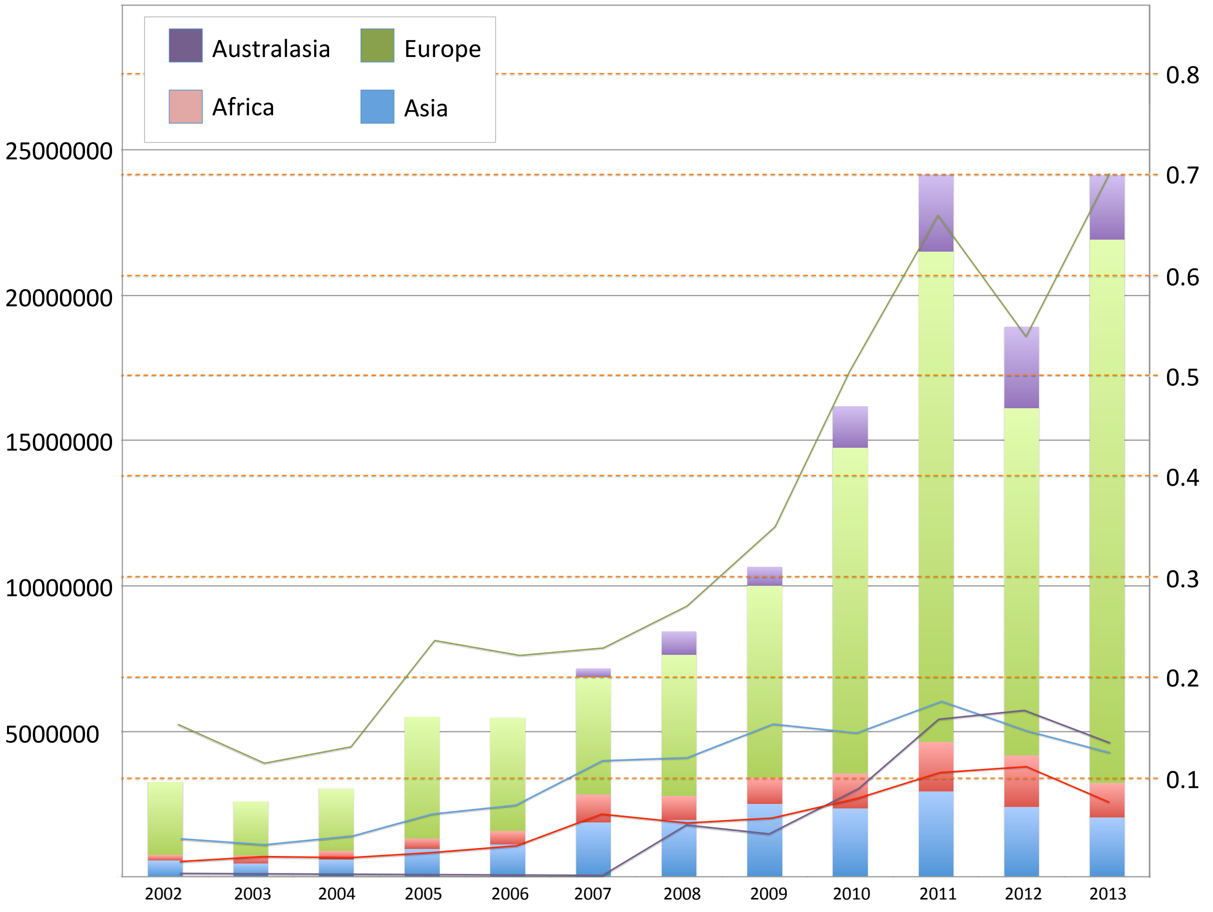

Suppl. Table 1: Helicoverpa armigera samples information including sampling sites (country and/or state), sample ID, number of individuals sampled per site (n), and associated mtDNA COI and Cyt b haplotypes. The 'Old World' continents are Asia, Africa, Europe, Australasia, and the 'New World' continent is South America. Host crops information from which larvae of various instar-stages were collected is also provided. Adult moths with unknown hosts were collected either by light traps or by pheromone traps.

	Country, State and/or closest city names, (Collection Date), Host crops.	n	**Sample ID**	**mtDNA COI Haplotpes**	**mtDNA Cyt *b* Haplotypes**
	**Asia**				
	**India**				
1	Punjab/Abohar January-2005)	5	**001**	Harm-10	Harm-01
2	Host: Chickpea		**002**	Harm-01	Harm-01
3			**003**	Harm-10	Harm-01
4			**004**	Harm-01	Harm-01
5			**005**	Harm-01	Harm-01
6	Punjab/Bhatinda (January-2005)	5	**006**	Harm-19	Harm-09
7	Host: Chickpea		**007**	Harm-17	Harm-11
8			**008**	Harm-01	Harm-01
9			**009**	Harm-23	Harm-08
10			**010**	Harm-01	Harm-01
11	Punjab/Mansa (January-2005)	5	**011**	Harm-01	Harm-01
12	Host: Chickpea		**012**	Harm-01	Harm-01
13			**013**	Harm-01	Harm-10
14			**014**	Harm-24	Harm-11
15			**015**	Harm-01	Harm-01
16	Maharashtra/Nagpur (January-2005)	11	**016**	Harm-01	Harm-09
17	Host: Unknown (adults)		**017**	Harm-01	Harm-11
18			**018**	Harm-01	Harm-09
19			**019**	Harm-01	Harm-01
20			**020**	Harm-01	Harm-01
21			**021**	Harm-01	Harm-01
22			**022**	Harm-01	Harm-01
23			**023**	Harm-27	Harm-01
24			**024**	Harm-03	Harm-02
25			**025**	Harm-01	Harm-01
26			**026**	Harm-01	Harm-01
27	Maharashtra/Yavatmal (July-2005)	10	**027**	Harm-01	Harm-11
28	Host: Egg Plant		**028**	Harm-01	Harm-01
29			**029**	Harm-03	Harm-02
30			**030**	Harm-01	Harm-01
31			**031**	Harm-15	Harm-01
32			**032**	Harm-01	Harm-01
33			**033**	Harm-17	Harm-11
34			**034**	Harm-01	Harm-01
35			**035**	Harm-03	Harm-03
36			**036**	Harm-01	Harm-01
37	Maharashtra/Hingoli (November-2004)	5	**037**	Harm-01	Harm-01
38	Host: Cotton		**038**	Harm-01	Harm-01

39			**039**	Harm-10	Harm-01
40			**040**	Harm-10	Harm-09
41			**041**	Harm-01	Harm-01
42	Andhra Pradesh/Prakasam (December-2004)	6	**042**	Harm-01	Harm-09
43	Host: Cotton		**043**	Harm-01	Harm-09
44			**044**	Harm-01	Harm-09
45			**045**	Harm-01	Harm-01
46			**046**	Harm-01	Harm-09
47			**047**	Harm-01	Harm-01
48	Tamil Nadu/Coimbatore (January-2005)	22	**048**	Harm-01	Harm-11
49	Host: Pigeonpea		**049**	Harm-06	Harm-01
50			**050**	Harm-10	Harm-01
51			**051**	Harm-09	Harm-11
52			**052**	Harm-01	Harm-01
53			**053**	Harm-01	Harm-01
54			**054**	Harm-03	Harm-02
55			**055**	Harm-03	Harm-02
56			**056**	Harm-01	Harm-25
57			**057**	Harm-01	Harm-11
58			**058**	Harm-01	Harm-12
59			**059**	Harm-03	Harm-02
60			**060**	Harm-03	Harm-02
61			**061**	Harm-03	Harm-02
62			**062**	Harm-01	Harm-12
63			**063**	Harm-01	Harm-11
64			**064**	Harm-01	Harm-11
65			**065**	Harm-03	Harm-02
66			**066**	Harm-06	Harm-01
67			**067**	Harm-01	Harm-01
68			**068**	Harm-01	Harm-01
69			**069**	Harm-01	Harm-11
70	Telangana/Karimnagar (October-2005)	10	**070**	Harm-01	Harm-01
71	Host: Cotton		**071**	Harm-01	Harm-01
72			**072**	Harm-01	Harm-01
73			**073**	Harm-02	Harm-01
74			**074**	Harm-01	Harm-01
75			**075**	Harm-01	Harm-01
76			**076**	Harm-32	Harm-01
77			**077**	Harm-01	Harm-01
78			**078**	Harm-01	Harm-01
79			**079**	Harm-01	Harm-01
80	Telangana/Warangal (October-2005)	11	**080**	Harm-32	Harm-01
81	Host: Cotton		**081**	Harm-33	Harm-08
82			**082**	Harm-01	Harm-01
83			**083**	Harm-33	Harm-08
84			**084**	Harm-32	Harm-01
85			**085**	Harm-32	Harm-01
86			**086**	Harm-33	Harm-08
87			**087**	Harm-33	Harm-08
88			**088**	Harm-01	Harm-01

89			**089**	Harm-01	Harm-08
90			**090**	Harm-01	Harm-01
	**Pakistan**				
91	Multan (Nov-2004)	10	**091**	Harm-01	Harm-01
92	Host: Cotton		**092**	Harm-01	Harm-09
93			**093**	Harm-06	Harm-01
94			**094**	Harm-01	Harm-09
95			**095**	Harm-01	Harm-01
96			**096**	Harm-01	Harm-09
97			**097**	Harm-01	Harm-09
98			**098**	Harm-07	Harm-01
99			**099**	Harm-01	Harm-01
100			**100**	Harm-01	Harm-09
	**China**				
101	Shandong (February-2005)	34	**101**	Harm-01	Harm-08
102	Host: Cotton		**102**	Harm-01	Harm-08
103			**103**	Harm-03	Harm-06
104			**104**	Harm-06	Harm-01
105			**105**	Harm-01	Harm-01
106			**106**	Harm-01	Harm-01
107			**107**	Harm-02	Harm-11
108			**108**	Harm-01	Harm-01
109			**109**	Harm-01	Harm-16
110			**110**	Harm-01	Harm-14
111			**111**	Harm-01	Harm-22
112			**112**	Harm-01	Harm-01
113			**113**	Harm-01	Harm-21
114			**114**	Harm-01	Harm-01
115			**115**	Harm-10	Harm-01
116			**116**	Harm-01	Harm-01
117			**117**	Harm-01	Harm-01
118			**118**	Harm-01	Harm-01
119			**119**	Harm-01	Harm-19
120			**120**	Harm-01	Harm-01
121			**121**	Harm-15	Harm-09
122			**122**	Harm-01	Harm-01
123			**123**	Harm-01	Harm-01
124			**124**	Harm-22	Harm-19
125			**125**	Harm-01	Harm-01
126			**126**	Harm-01	Harm-01
127			**127**	Harm-03	Harm-07
128			**128**	Harm-01	Harm-01
129			**129**	Harm-21	Harm-01
130			**130**	Harm-01	Harm-20
131			**131**	Harm-01	Harm-09
132			**132**	Harm-01	Harm-01
133			**133**	Harm-10	Harm-01
134			**134**	Harm-01	Harm-01
	**Africa**				
135	Burkina Faso	35	**135**	Harm-01	Harm-01
136	Kenedougou (March-2003)		**136**	Harm-01	Harm-01

137	Host: Tomato		**137**	Harm-01	Harm-01
138			**138**	Harm-01	Harm-01
139			**139**	Harm-01	Harm-11
140			**140**	Harm-01	Harm-08
141			**141**	Harm-10	Harm-01
142			**142**	Harm-01	Harm-01
143			**143**	Harm-01	Harm-01
144			**144**	Harm-03	Harm-02
145			**145**	Harm-01	Harm-01
146			**146**	Harm-01	Harm-01
147			**147**	Harm-01	Harm-01
148			**148**	Harm-16	Harm-23
149			**149**	Harm-01	Harm-01
150			**150**	Harm-14	Harm-01
151			**151**	Harm-01	Harm-01
152			**152**	Harm-02	Harm-01
153			**153**	Harm-01	Harm-08
154			**154**	Harm-02	Harm-01
155			**155**	Harm-01	Harm-11
156			**156**	Harm-01	Harm-01
157			**157**	Harm-01	Harm-01
158			**158**	Harm-01	Harm-01
159			**159**	Harm-01	Harm-01
160			**160**	Harm-10	Harm-01
161			**161**	Harm-14	Harm-01
162			**162**	Harm-02	Harm-01
163			**163**	Harm-01	Harm-01
164			**164**	Harm-01	Harm-01
165			**165**	Harm-01	Harm-01
166			**166**	Harm-17	Harm-01
167			**167**	Harm-01	Harm-01
168			**168**	Harm-01	Harm-08
169			**169**	Harm-01	Harm-11
170	Uganda	24	**170**	Harm-04	Harm-04
171	Kampala (November-2005)		**171**	Harm-02	Harm-01
172	Host: Cotton		**172**	Harm-02	Harm-15
173			**173**	Harm-01	Harm-01
174			**174**	Harm-11	Harm-01
175			**175**	Harm-01	Harm-01
176			**176**	Harm-01	Harm-09
177			**177**	Harm-01	Harm-08
178			**178**	Harm-12	Harm-01
179			**179**	Harm-01	Harm-08
180			**180**	Harm-01	Harm-01
181			**181**	Harm-01	Harm-01
182			**182**	Harm-01	Harm-08
183			**183**	Harm-01	Harm-08
184			**184**	Harm-01	Harm-01
185			**185**	Harm-03	Harm-02
186			**186**	Harm-01	Harm-01
187			**187**	Harm-25	Harm-01
188			**188**	Harm-19	Harm-09
189			**189**	Harm-01	Harm-01

190			**190**	Harm-26	Harm-08
191			**191**	Harm-01	Harm-01
192			**192**	Harm-01	Harm-01
193			**193**	Harm-11	Harm-01
194	Senegal	11	**194**	Harm-01	Harm-01
195	Noto (2005)		**195**	Harm-02	Harm-11
196	Host: Tomato		**196**	Harm-01	Harm-27
197			**197**	Harm-01	Harm-28
198			**198**	Harm-34	Harm-01
199			**199**	Harm-01	Harm-01
200			**200**	Harm-01	Harm-08
201			**201**	Harm-02	Harm-11
202			**202**	Harm-01	Harm-11
203			**203**	Harm-01	Harm-11
204			**204**	Harm-35	Harm-01
205	Ghana	4	**205**	Harm-36	Harm-20
206	Tamale (June-2014)		**206**	Harm-01	Harm-01
207	Host: Cowpea		**207**	Harm-37	Harm-01
208			**208**	Harm-01	Harm-01
209	Madagascar	14	**209**	Harm-38	Harm-01
210	Antsirabe (2006)		**210**	Harm-01	Harm-01
211	Host: Corn		**211**	Harm-39	Harm-01
212			**212**	Harm-01	Harm-08
213			**213**	Harm-01	Harm-01
214			**214**	Harm-02	Harm-08
215			**215**	Harm-01	Harm-01
216			**216**	Harm-40	Harm-01
217			**217**	Harm-01	Harm-11
218			**218**	Harm-33	Harm-01
219			**219**	Harm-01	Harm-01
220			**220**	Harm-01	Harm-01
221			**221**	Harm-06	Harm-01
222			**222**	Harm-01	Harm-08
223	Cameroon	13	**223**	Harm-01	Harm-08
224	Dakar (2005)		**224**	Harm-01	Harm-01
225	Host: Tomato		**225**	Harm-01	Harm-01
226			**226**	Harm-01	Harm-01
227			**227**	Harm-01	Harm-01
228			**228**	Harm-01	Harm-01
229			**229**	Harm-01	Harm-29
230			**230**	Harm-41	Harm-08
231			**231**	Harm-01	Harm-29
232			**232**	Harm-01	Harm-01
233			**233**	Harm-01	Harm-01
234			**234**	Harm-21	Harm-01
235			**235**	Harm-01	Harm-08
236	Chad	6	**236**	Harm-01	Harm-01
237	Pala (2006)		**237**	Harm-42	Harm-01
238	Host: Cotton		**238**	Harm-43	Harm-01

239			**239**	Harm-01	Harm-11
240			**240**	Harm-49	Harm-08
241			**241**	Harm-01	Harm-01
	**Australasia**				
	**Australia**				
242	Victoria/Dalmore (April-2005)	24	**242**	Harm-02	Harm-01
243	Host: Corn		**243**	Harm-01	Harm-01
244			**244**	Harm-02	Harm-01
245			**245**	Harm-05	Harm-11
246			**246**	Harm-08	Harm-01
247			**247**	Harm-02	Harm-24
248			**248**	Harm-01	Harm-13
249			**249**	Harm-02	Harm-17
250			**250**	Harm-01	Harm-01
251			**251**	Harm-01	Harm-01
252			**252**	Harm-05	Harm-01
253			**253**	Harm-01	Harm-01
254			**254**	Harm-01	Harm-01
255			**255**	Harm-02	Harm-01
256			**256**	Harm-02	Harm-17
257			**257**	Harm-04	Harm-04
258			**258**	Harm-01	Harm-01
259			**259**	Harm-01	Harm-01
260			**260**	Harm-01	Harm-01
261			**261**	Harm-02	Harm-17
262			**262**	Harm-01	Harm-06
263			**263**	Harm-01	Harm-01
264			**264**	Harm-02	Harm-11
265			**265**	Harm-01	Harm-25
266	Victoria/Orbost (April-2005)	22	**266**	Harm-01	Harm-01
267	Host: Corn		**267**	Harm-01	Harm-01
268			**268**	Harm-05	Harm-01
269			**269**	Harm-05	Harm-11
270			**270**	Harm-01	Harm-01
271			**271**	Harm-01	Harm-01
272			**272**	Harm-31	Harm-01
273			**273**	Harm-01	Harm-01
274			**274**	Harm-03	Harm-01
275			**275**	Harm-01	Harm-05
276			**276**	Harm-01	Harm-01
277			**277**	Harm-01	Harm-18
278			**278**	Harm-01	Harm-17
279			**279**	Harm-01	Harm-06
280			**280**	Harm-01	Harm-08
281			**281**	Harm-29	Harm-01
282			**282**	Harm-03	Harm-01
283			**283**	Harm-13	Harm-01
284			**284**	Harm-01	Harm-01
285			**285**	Harm-05	Harm-01
286			**286**	Harm-01	Harm-01
287			**287**	Harm-02	Harm-01

288	Victoria/Werribee (January-2001)	10	**288**	Harm-30	Harm-01
289	Host: Unknown (adults)		**289**	Harm-10	Harm-01
290			**290**	Harm-01	Harm-01
291			**291**	Harm-28	Harm-01
292			**292**	Harm-13	Harm-01
293			**293**	Harm-18	Harm-01
294			**294**	Harm-01	Harm-01
295			**295**	Harm-02	Harm-01
296			**296**	Harm-20	Harm-01
297			**297**	Harm-01	Harm-26
	**NEW ZEALAND**				
298	Auckland/Pukekohe (June-2004)	9	**298**	Harm-01	Harm-01
299	Host: Unknown (adults)		**299**	Harm-01	Harm-01
300			**300**	Harm-05	Harm-01
301			**301**	Harm-01	Harm-01
302			**302**	Harm-47	Harm-01
303			**303**	Harm-02	Harm-01
304			**304**	Harm-01	Harm-01
305			**305**	Harm-01	Harm-01
306			**306**	Harm-01	Harm-01
	**Europe**				
	**France**				
307	Montpellier (2013)	3	**307**	Harm-33	Harm-01
308	Host: Medicago		**308**	Harm-33	Harm-01
309			**309**	Harm-33	Harm-01
310	French Corsica (July-2013)	14	**310**	Harm-01	Harm-01
311	Host: Medicago		**311**	Harm-01	Harm-08
312			**312**	Harm-50	Harm-30
313			**313**	Harm-01	Harm-01
314			**314**	Harm-51	Harm-31
315			**315**	Harm-01	Harm-01
316			**316**	Harm-48	Harm-32
317			**317**	Harm-03	Harm-05
318			**318**	Harm-01	Harm-33
319			**319**	Harm-01	Harm-34
320			**320**	Harm-01	Harm-08
321			**321**	Harm-51	Harm-31
322			**322**	Harm-01	Harm-08
323			**323**	Harm-01	Harm-01
	**Spain**				
324	Seville (September-2013)	6	**324**	Harm-01	Harm-01
325	Host: Medicago		**325**	Harm-52	Harm-09
326			**326**	Harm-01	Harm-01
327			**327**	Harm-53	Harm-11
328			**328**	Harm-52	Harm-01
329			**329**	Harm-01	Harm-01
	**Old World Total**	329			
	**South America**				
	**BRAZIL**				

1	Goiás/Palmeiras de Goiás (August-2013)	10	**330**	Harm-01	Harm-35
2	Host: Tomato		**331**	Harm-01	Harm-21
3			**332**	Harm-01	Harm-08
4			**333**	Harm-01	Harm-08
5			**334**	Harm-01	Harm-35
6			**335**	Harm-01	Harm-11
7			**336**	Harm-01	Harm-35
8			**337**	Harm-01	Harm-35
9			**338**	Harm-01	Harm-08
10			**339**	Harm-01	Harm-08
11	Goiás/Palmeiras de Goiás (2013)	8	**340**	Harm-21	Harm-01
12	Host: Millet		**341**	Harm-01	Harm-01
13			**342**	Harm-01	Harm-01
14			**343**	Harm-01	Harm-01
15			**344**	Harm-01	Harm-01
16			**345**	Harm-01	Harm-01
17			**346**	Harm-01	Harm-01
18	Goiás/Brasília/Planaltina (Distrito Federal) (2013)	8	**347**	Harm-35	Harm-01
19	Host: Bean		**348**	Harm-02	Harm-01
20			**349**	Harm-01	Harm-08
21			**350**	Harm-01	Harm-01
22			**351**	Harm-01	Harm-01
23			**352**	Harm-02	Harm-01
24			**353**	Harm-01	Harm-01
25			**354**	Harm-01	Harm-01
26			**355**	Harm-01	Harm-01
27	Goiás/Morrinhos (August-2013)	9	**356**	Harm-02	Harm-01
28	Host: Tomato		**357**	Harm-01	Harm-01
29			**358**	Harm-01	Harm-08
30			**359**	Harm-01	Harm-11
31			**360**	Harm-01	Harm-08
32			**361**	Harm-01	Harm-08
33			**362**	Harm-01	Harm-01
34			**363**	Harm-04	Harm-02
35			**364**	Harm-54	Harm-01
36	Minas Gerais/Patos de Minas (April-2013)	7	**365**	Harm-02	Harm-01
37	Host: Cotton		**366**	Harm-01	Harm-01
38			**367**	Harm-02	Harm-01
39			**368**	Harm-01	Harm-08
40			**369**	Harm-01	Harm-01
41			**370**	Harm-02	Harm-01
42			**371**	Harm-01	Harm-01
43	Goiás/Brazabrantes (August-2013)	11	**372**	Harm-02	Harm-01
44	Host: Tomato		**373**	Harm-01	Harm-08
45			**374**	Harm-01	Harm-01
46			**375**	Harm-01	Harm-36
47			**376**	Harm-01	Harm-04
48			**377**	Harm-01	Harm-01
49			**378**	Harm-01	Harm-11

50			**379**	Harm-46	Harm-01
51			**380**	Harm-01	Harm-01
52			**381**	Harm-01	Harm-04
53			**382**	Harm-01	Harm-01
54	Paraná/Ivaiporã (August-2013)	3	**383**	Harm-01	Harm-08
55	Host: Wheat		**384**	Harm-01	Harm-01
56			**385**	Harm-01	Harm-08
57	Bahia/Correntina (April-2013)	1	**386**	Harm-01	Harm-01
	Host: cotton				
58	Maranhão/Alto Parnaíba (April-2013)	2	**387**	Harm-01	Harm-01
59	Host: cotton		**388**	Harm-04	Harm-04
60	Mato Grosso/PVA do Leste (May-2013)	7	**389**	Harm-01	Harm-08
61	Host: Cotton		**390**	Harm-01	Harm-01
62			**391**	Harm-01	Harm-11
63			**392**	Harm-03	Harm-05
64			**393**	Harm-01	Harm-01
65			**394**	Harm-01	Harm-01
66			**395**	Harm-01	Harm-08
67	Mato Grosso/Pedra Preta (April-2013)	3	**396**	Harm-01	Harm-01
68	Host: Cotton		**397**	Harm-01	Harm-08
69			**398**	Harm-01	Harm-01
70	Mato Grosso/PVA do Leste (June-2013)	9	**399**	Harm-03	Harm-05
71	Host: Cotton		**400**	Harm-02	Harm-08
72			**401**	Harm-01	Harm-01
73			**402**	Harm-03	Harm-05
74			**403**	Harm-01	Harm-01
75			**404**	Harm-01	Harm-11
76			**405**	Harm-01	Harm-08
77			**406**	Harm-02	Harm-01
78			**407**	Harm-01	Harm-11
79	Mato Grosso/Pedra Preta (March-2013)	4	**408**	Harm-01	Harm-01
80	Host: Cotton		**409**	Harm-01	Harm-01
81			**410**	Harm-44	Harm-01
82			**411**	Harm-44	Harm-01
83	Mato Grosso (June-2013)	2	**412**	Harm-01	Harm-01
84	Host: Pearl Millet		**413**	Harm-01	Harm-01
85	Mato Grosso/Ipiranga do Norte (June-2013)	3	**414**	Harm-01	Harm-01
86	Host: Cotton		**415**	Harm-54	Harm-01
87			**416**	Harm-01	Harm-01
88	Mato Grosso/Campo Verde (May-2013)	1	**417**	Harm-01	Harm-11
	Host: Corn				
89	Mato Grosso/Campo Verde (June-2013)	6	**418**	Harm-01	Harm-01
90	Host: Cotton		**419**	Harm-01	Harm-01

91			**420**	Harm-01	Harm-01
92			**421**	Harm-01	Harm-01
93			**422**	Harm-01	Harm-11
94			**423**	Harm-01	Harm-01
95	Mato Grosso (June-2013)	6	**424**	Harm-01	Harm-01
96	Host: Sunflower		**425**	Harm-01	Harm-01
97			**426**	Harm-01	Harm-01
98			**427**	Harm-01	Harm-01
99			**428**	Harm-01	Harm-11
100			**429**	Harm-01	Harm-01
101	Mato Grosso/Pedra Preta (June-2013)	1	**430**	Harm-02	Harm-01
	Host: Cotton				
102	Mato Grosso/Guiratinga (June-2013)	3	**431**	Harm-01	Harm-01
103	Host: Cotton		**432**	Harm-10	Harm-01
104			**433**	Harm-01	Harm-01
105	Mato Grosso/Rondonópolis (June-2013)	3	**434**	Harm-01	Harm-08
106	Host: Cotton		**435**	Harm-01	Harm-01
107			**436**	Harm-01	Harm-08
108	Mato Grosso/Sapezal (June-2013)	7	**437**	Harm-01	Harm-08
109	Host: Unknown (adult moths)		**438**	Harm-15	Harm-01
110			**439**	Harm-01	Harm-01
111			**440**	Harm-01	Harm-08
112			**441**	Harm-45	Harm-08
113			**442**	Harm-01	Harm-01
114			**443**	Harm-15	Harm-01
	**Old World Total**	114			

Suppl. Table 2: Global H. armigera mtDNA COI and Cyt b haplotypes. Matched unique mtDNA COI-Cytb haplotypes between Brazil and Old World countries are in red colour. Unmatched unique Brazilian haplotypes are in Blue. Filled cells indicates haplotypes detected in individual countries. Haplotype codes are as referred in the main text and are provided for (COI, Cytb). ISO country codes are:NZL (New Zealand), AUS (Australia), CHN (China), IND (India), PAK (Pakistan), FRA (France), ESP (Spain), GHA (Ghana), UGA (Uganda), BFA (Burkina Faso), TCD (Chad), MDG (Madagascar), CMR (Cameroon), SEN (Senegal), BRA (Brazil). Note that 21 new mtDNA COI (GenBank: KX494879-KX494899) and 10 new mtDNA Cyt b haplotypes (GenBank: KX494900-KX494909) were found in Senegal (n_COI=2; n_Cytb=2), Ghana (n_COI=2), Madagascar (n_COI=3), Chad (n_COI=3); Cameroon (n_COI=1; n_Cytb=1); Spain (n_COI=3), French Corsica (n_COI=3; n_Cytb=5), New Zealand (n_COI=1), and Brazil (n_COI=5; n_Cytb=2), and seven haplotypes were found in Brazil as well as in more than one other country (i.e., Hap0101 in New Zealand, Australia, China, India, Pakistan, France, Spain, Cameroon, Uganda, Ghana, Madagascar, Chad, Burkina Faso and Senegal; Hap0108 in Australia, France, China, India, Uganda, Burkina Faso, Madagascar, Cameroon and Senegal; Hap0111 in Madagascar, Cameroon, Burkina Faso, Chad and India; Hap0201 in New Zealand, Australia, India, Burkina Faso and Uganda; Hap0404 in Uganda and Australia; Hap1001 in Australia, China, India and Burkina Faso; and Hap2101 in China and Cameroon).

	Haplotype codes	NZL	AUS	CHN	IND	PAK	FRA	ESP	GHA	UGA	BFA	TCD	MDG	CMR	SEN	BRA	COI Haplotypes	Cyt *b* Haplotypes	COI Haplotype GenBank Acc. No.	Cyt *b* Haplotypes GenBank Acc. No.
1	Hap0101																COI-Harm-01	Harm-01	EF116226	EF410022
2	Hap0104																COI-Harm-01	Harm-04	EF116226	EF410023
3	Hap0105																COI-Harm-01	Harm-05	EF116226	EF410024
4	Hap0106																COI-Harm-01	Harm-06	EF116226	EF410025
5	Hap0108																COI-Harm-01	Harm-08	EF116226	EF410027
6	Hap0109																COI-Harm-01	Harm-09	EF116226	EF410028
7	Hap0110																COI-Harm-01	Harm-10	EF116226	EF410029
8	Hap0111																COI-Harm-01	Harm-11	EF116226	EF410030
9	Hap0112																COI-Harm-01	Harm-12	EF116226	EF410031
10	Hap0113																COI-Harm-01	Harm-13	EF116226	EF410032
11	Hap0114																COI-Harm-01	Harm-14	EF116226	EF410033
12	Hap0116																COI-Harm-01	Harm-16	EF116226	EF410035
13	Hap0117																COI-Harm-01	Harm-17	EF116226	EF410036
14	Hap0118																COI-Harm-01	Harm-18	EF116226	EF410037
15	Hap0119																COI-Harm-01	Harm-19	EF116226	EF410038
16	Hap0120																COI-Harm-01	Harm-20	EF116226	EF410039
17	Hap0121																COI-Harm-01	Harm-21	EF116226	EF410040
18	Hap0122																COI-Harm-01	Harm-22	EF116226	EF410041
19	Hap0125																COI-Harm-01	Harm-25	EF116226	EF410044
20	Hap0126																COI-Harm-01	Harm-26	EF116226	EF410045
21	Hap0127																COI-Harm-01	Harm-27	EF116226	KX494900
22	Hap0128																COI-Harm-01	Harm-28	EF116226	KX494901
23	Hap0129																COI-Harm-01	Harm-29	EF116226	KX494902
24	Hap0133																COI-Harm-01	Harm-33	EF116226	KX494906
25	Hap0134																COI-Harm-01	Harm-34	EF116226	KX494907
26	Hap0135																COI-Harm-01	Harm-35	EF116226	KX494908
27	Hap0136																COI-Harm-01	Harm-36	EF116226	KX494909
28	Hap0201																COI-Harm-02	Harm-01	EF116227	EF410020
29	Hap0208																COI-Harm-02	Harm-08	EF116227	EF410027
30	Hap0211																COI-Harm-02	Harm-11	EF116227	EF410030
31	Hap0215																COI-Harm-02	Harm-15	EF116227	EF410034
32	Hap0217																COI-Harm-02	Harm-17	EF116227	EF410036
33	Hap0224																COI-Harm-02	Harm-24	EF116227	EF410043
34	Hap0301																COI-Harm-03	Harm-01	EF116228	EF410020
35	Hap0302																COI-Harm-03	Harm-02	EF116228	EF410021
36	Hap0303																COI-Harm-03	Harm-03	EF116228	EF410022
37	Hap0305																COI-Harm-03	Harm-05	EF116228	EF410024
38	Hap0306																COI-Harm-03	Harm-06	EF116228	EF410025
39	Hap0307																COI-Harm-03	Harm-07	EF116228	EF410026
40	Hap0402																COI-Harm-04	Harm-02	EF116229	EF410021
41	Hap0404																COI-Harm-04	Harm-04	EF116229	EF410023
42	Hap0501																COI-Harm-05	Harm-01	EF116230	EF410020
43	Hap0511																COI-Harm-05	Harm-11	EF116230	EF410030
44	Hap0601																COI-Harm-06	Harm-01	EF116231	EF410020
45	Hap0701																COI-Harm-07	Harm-01	EF116232	EF410020
46	Hap0801																COI-Harm-08	Harm-01	EF116233	EF410020
47	Hap0911																COI-Harm-09	Harm-11	EF116234	EF410030
48	Hap1001																COI-Harm-10	Harm-01	EF116235	EF410020
49	Hap1009																COI-Harm-10	Harm-09	EF116235	EF410028
50	Hap1101																COI-Harm-11	Harm-01	EF116236	EF410020
51	Hap1201																COI-Harm-12	Harm-01	EF116237	EF410020
52	Hap1301																COI-Harm-13	Harm-01	EF116238	EF410020
53	Hap1401																COI-Harm-14	Harm-01	EF116239	EF410020
54	Hap1501																COI-Harm-15	Harm-01	EF116240	EF410020
55	Hap1509																COI-Harm-15	Harm-09	EF116240	EF410028
56	Hap1623																COI-Harm-16	Harm-23	EF116241	EF410042
57	Hap1701																COI-Harm-17	Harm-01	EF116242	EF410020
58	Hap1711																COI-Harm-17	Harm-11	EF116242	EF410030
59	Hap1801																COI-Harm-18	Harm-01	EF116243	EF410020
60	Hap1909																COI-Harm-19	Harm-09	EF116244	EF410028
61	Hap2001																COI-Harm-20	Harm-01	EF116245	EF410020
62	Hap2101																COI-Harm-21	Harm-01	EF116246	EF410020
63	Hap2219																COI-Harm-22	Harm-19	EF116247	EF410038
64	Hap2308																COI-Harm-23	Harm-08	EF116248	EF410027
65	Hap2411																COI-Harm-24	Harm-11	EF116249	EF410030
66	Hap2501																COI-Harm-25	Harm-01	EF116250	EF410020
67	Hap2608																COI-Harm-26	Harm-08	EF116251	EF410027
68	Hap2701																COI-Harm-27	Harm-01	EF116252	EF410020
69	Hap2801																COI-Harm-28	Harm-01	EF116253	EF410020
70	Hap2901																COI-Harm-29	Harm-01	EF116254	EF410020
71	Hap3001																COI-Harm-30	Harm-01	EF116255	EF410020
72	Hap3101																COI-Harm-31	Harm-01	EF116256	EF410020
73	Hap3201																COI-Harm-32	Harm-01	EF116257	EF410020
74	Hap3301																COI-Harm-33	Harm-01	EF116258	EF410020
75	Hap3308																COI-Harm-33	Harm-08	EF116258	EF410027
76	Hap3401																COI-Harm-34	Harm-01	KX494879	EF410020
77	Hap3501																COI-Harm-35	Harm-01	KX494880	EF410020
78	Hap3620																COI-Harm-36	Harm-20	KX494881	EF410039
79	Hap3701																COI-Harm-37	Harm-01	KX494882	EF410020
80	Hap3801																COI-Harm-38	Harm-01	KX494883	EF410020
81	Hap3901																COI-Harm-39	Harm-01	KX494884	EF410020
82	Hap4001																COI-Harm-40	Harm-01	KX494885	EF410020
83	Hap4108																COI-Harm-41	Harm-08	KX494886	EF410027
84	Hap4201																COI-Harm-42	Harm-01	KX494887	EF410020
85	Hap4301																COI-Harm-43	Harm-01	KX494888	EF410020
86	Hap4401																COI-Harm-44	Harm-01	KX494889	EF410020
87	Hap4508																COI-Harm-45	Harm-08	KX494890	EF410027
88	Hap4601																COI-Harm-46	Harm-01	KX494891	EF410020
89	Hap4701																COI-Harm-47	Harm-01	KX494892	EF410020
90	Hap4832																COI-Harm-48	Harm-32	KX494893	KX494905
91	Hap4908																COI-Harm-49	Harm-08	KX494894	EF410027
92	Hap5030																COI-Harm-50	Harm-30	KX494895	KX494903
93	Hap5131																COI-Harm-51	Harm-31	KX494896	KX494904
94	Hap5201																COI-Harm-52	Harm-01	KX494897	EF410020
95	Hap5209																COI-Harm-52	Harm-09	KX494897	EF410028
96	Hap5311																COI-Harm-53	Harm-11	KX494898	EF410030
97	Hap5401																COI-Harm-54	Harm-01	KX494899	EF410020

Suppl. Table 3: Brazil importation of agricultural and horticultural commodities (in financial volume of $USD ,000) between 2002 to 2013 as represented by the Harmonized System (HS) code 06 for Other Live Plants (including hs92_product_ID: 0602, 060210, 060310; and 060491) from global destinations. International Organisation for Standardisation (ISO) three-letters country codes are used. Countries are grouped by continents (Africa = yellow; Asia = red; Europe = purple; North America = blue; South America = green; Australasia = orange). 'nes' = not elsewhere specified.

Where does Brazil import Other Live Plants from 2002-2013

Other Live Plants	2002	2003	2004	2005	2006	2007	2008	2009	2010	2011	2012	2013
hs92_product_id: 0602	ZAF	EGY	IDN	CHN	EGY	ZAF	ZAF	ZAF	ZAF	ZAF	ZAF	ZAF
Brazil Import from:	16598.36	159367.87	4598	1182.52	7162	21341	3333.02	172026.92	404196	270970.33	68967.2	112341.23
	CHN	ISR	ISR	IDN	CHN	IND	ISR	HKG	HKG	HKG	CHN	CHN
	3774.23	35786.67	118332.99	4185	14389.95	32280	250817.26	25802	59002	31440	10253.71	26184.75
	ISR	JPN	JPN	ISR	IDN	ISR	JPN	ISR	ISR	ISR	ISR	HKG
	134505.49	1403.21	1342.37	406785.15	2325	282196.59	72277.21	53214.6	76010.81	334684.56	816486.39	79560
	JPN	THA	KOR	JPN	ISR	JPN	KOR	JPN	JPN	JPN	JPN	ISR
	9508.88	13798.72	4950	18495.37	91146.68	16837.22	1875	173266.31	208425.42	295619.37	394929.75	283157.78
	THA	DEU	THA	KOR	JPN	THA	THA	THA	THA	THA	THA	JPN
	19871.16	1138.1	30393.22	4758.38	10909.76	161300.1	199724.93	461596.91	655836.93	1024733.01	2316708.63	321689.26
	ESP	ESP	XXB	THA	MYS	XXB	XXB	XXB	XXB	XXB	XXB	MYS
	6944.05	3691.53	4501.46	40181.48	2826	16216.56	77447.21	81697.41	96155.77	102705.9	160239.43	4056.91
	FRA	FRA	DEU	XXB	THA	DEU	DEU	DEU	DEU	DEU	DEU	THA
	1873393.37	813114.25	3276.21	7712.05	48961.62	36926.79	38498.4	45734.98	77910.31	87682.82	113267.11	3093462.19
	ITA	ITA	DNK	BLX	XXB	ESP	ESP	ESP	GBR	ESP	ESP	XXB (Other Asia)
	1264837.8	420555.84	51398	3001	13874.51	7578.6	36270.46	13853.49	4553	26075.78	11528.89	115722.14
	NLD	NLD	ESP	DEU	DEU	ITA	ITA	ITA	ITA	ITA	ITA	DEU
	1083423.15	1572152.38	4535.31	24581.3	34352.17	472836.97	303798.44	287387.51	502014.47	1195562.44	395455.31	52304.48
	PRT	PRT	FRA	ESP	DNK	NLD	NLD	NLD	NLD	NLD	NLD	ITA
	51976.71	72465.49	1011121.63	52802.04	18487	2934471.98	4164514.36	4303925.84	5078519.63	7240735.48	8548119.3	338314.06
	CRI	CRI	ITA	ITA	ESP	PRT	PRT	CRI	CRI	CRI	PRT	NLD
	972733.17	427140.16	310696.15	222401.37	22576.37	8343.91	7414	310809.28	47926.21	65842	12217	9805777.95
	HND	HND	NLD	NLD	ITA	CRI	USA	USA	USA	USA	CRI	BRB
	34371.01	96793.25	1664239.46	1585104.31	353156.64	137151.06	40182.09	159952.52	433646.3	417388.68	74069.67	13000
	USA	USA	PRT	PRT	NLD	USA	AUS	ARG	ARG	ARG	USA	USA
	30743.93	65000	18820.99	7389	2470112.1	32337.83	5833.29	782801.49	1383849	1979914.21	708463.25	360892.48
	ARG	AUS	CRI	CRI	PRT	ARG	ARG	BOL	BOL	BOL	ARG	ARG
	179176	8186	379051.38	215850	11064.62	441703.18	605033.14	1212938.76	1056283	1390659.24	1841506	1266006.75
	CHL	ARG	HND	USA	CRI	CHL	BOL	CHL	CHL	CHL	BOL	BOL
	489985.87	110451.45	23420.61	185405.17	356954.7	1345405.12	391959.99	1967923.28	3152535.8	4027243.4	1137352.22	1352996.89
	COL	CHL	USA	AUS	USA	COL	CHL	COL	COL	COL	CHL	CHL
	20609.69	404676.37	29283.23	18922.55	6998.71	13788.43	1555835.43	30065.89	48942	128551	5794341.36	5362215.3
	URY	COL	AUS	NZL	AUS	ECU	COL	URY	ECU	ECU	COL	COL
	37745.71	6191.14	24464.55	1793	3172.11	3438	76960	19596	2789.41	4497.57	14962.38	80467.4
		ECU	ARG	ARG	ARG	URY	URY		URY	URY	ECU	ECU
		1847	103499.62	123509.68	326279.16	202275	21336		14000	58320	14425	12872.17
		URY	CHL	CHL	CHL						URY	PER
		3783	519126.14	683084	1286782.25						112902.8	1710
			COL	COL	COL							URY
			16137.84	23252	14800							8000
			URY	URY	ECU							
			31703.27	36322.48	4264.94							
					URY							
					50830							

hs92_product_id: 0602	2002	2003	2004	2005	2006	2007	2008	2009	2010	2011	2012	2013
Asia	167659.76	50988.6	164118.04	483299.95	184433.52	508830.47	602141.61	795577.23	1095430.93	1789182.84	3698617.91	3923833.03
Africa	16598.36	159367.87			7162	21341	3333.02	172026.92	404196	270970.33	68967.2	112341.23
EU	4228598.37	2810652.1	3045266.76	1887890.02	2898684.28	3451814.34	4543081.66	4650901.82	5662997.41	8550056.52	9068370.61	10196396.49
Australasia		8186	24464.55	20715.55	3172.11		5833.29					

Where does Brazil import Cuttings and slips, not rooted from 2002-2013

cuttings&slips_not rooted	2002	2003	2004	2005	2006	2007	2008	2009	2010	2011	2012	2013
hs92_product_id: 060210	ISR	ZAF	ITA	ISR	EGY	ISR	ISR	ZAF	ZAF	ZAF	ISR	HKG
Brazil Import from:	3000	76342	5184	3000	7162	93000	83000	84378	190995	37203	409956.99	79560
	JPN	ISR	NLD	JPN	ISR	DEU	DEU	HKG	HKG	HKG	DEU	ISR
	1903	3000	537451	5392	4000	7378.87	13891.93	25802	59002	31440	28555	82000
	FRA	DEU	PRT	DEU	MYS	ESP	ITA	ISR	DEU	ISR	ITA	DEU
	473931	1138.1	9937	13000	2826	2839	28589.88	15000	43726	45372	36354.96	17556.33
	ITA	FRA	CRI	NLD	DEU	ITA	NLD	DEU	ITA	JPN	NLD	ITA
	213481	61121	92400	614372	15000	62257	959301	13797.97	1848	3102	147772	32619
	NLD	ITA	USA	PRT	DNK	NLD	PRT	NLD	NLD	DEU	USA	NLD
	394924	104327	16728	7389	18487	1190239	7414	397655	64989	25381.28	240495	135211
	USA	NLD	ARG	CRI	ITA	COL	BOL	USA	USA	ITA	ARG	USA
	7650	651473	9857.04	12000	5391	11925	290476	4400	93611	27790.47	3120	30648
	COL	COL	COL	COL	NLD		COL	BOL	ARG	NLD	BOL	BOL
	14313	4493	11608	11664	1134476		38235	731804	8250	65359	337178	442857
					PRT			COL	BOL	USA	COL	COL
					3669.52			25945	640016	133112	12919	27881.4
					COL				COL	BOL		
					7300				32425	546155		
										COL		

hs92_product_id: 060210	2002	2003	2004	2005	2006	2007	2008	2009	2010	2011	2012	2013
Asia	4903	3000	0	8392	6826	93000	83000	40802	59002	79914	409956.99	161560
Africa	0	76342	0	0	7162	0	0	84378	190995	37203	0	0
EU	1082336	818059.1	552572	634761	1177023.52	1262713.87	1009196.81	411452.97	110563	118530.75	212681.96	185386.33
NZ	0	0	0	0	0	0	0	0	0	0	0	0

Where does Brazil import Cut flowers and flower buds for bouquets, etc., fresh from 2002-2013

Cut Flowers_fresh	2002	2003	2004	2005	2006	2007	2008	2009	2010	2011	2012	2013
hs92_ProductID: 060310	IND	IRL	IND	IND	ETH	DEU	ETH	NGA	NGA	EGY	JPN	NLD
Brazil Import from:	30831	9327	1259	2562	58021	3000	1056	2687	2329	23510	11914	26835.52
	NLD	NLD	NLD	ITA	IND	NLD	NLD	THA	JPN	IND	THA	COL
	1151.17	4524	8979.22	2711.63	1982	30121.88	34167.08	14034	99043	6313	40000	5151779.79
	BOL	CHL	COL	NLD	DEU	NZL	COL	COL	THA	THA	NLD	ECU
	2130.83	2500.15	632871.17	22541.22	2656.16	2401	2202276.84	2535153.88	14113	9344	45295.18	3757027.52

	2002	2003	2004	2005	2006	2007	2008	2009	2010	2011	2012	2013
	CHL	COL	ECU	CHL	ITA	COL	ECU	ECU	NLD	NLD	COL	
	4756.85	775414.17	9942.68	2340	4564.58	1409857.81	183379.44	345358.34	76294.48	107834.17	4365294.78	
	COL	ECU		COL	NLD	ECU			COL	COL	ECU	
	1071273.23	42321.51		947613.96	51241.33	201991.33			3360093.25	4731481	3997678.68	
	ECU			ECU	CHL				ECU	ECU		
	181623.4			37561.3	2340				1426567.71	3218270.94		
					COL							
					1286370.48							
					ECU							
					108659.54							

hs92_ProductID: 060310	2002	2003	2004	2005	2006	2007	2008	2009	2010	2011	2012	2013
Asia	30831	0	1259	2562	1982	0	0	14034	113156	15657	51914	0
Africa	0	0	0	0	58021	0	1056	2687	2329	23510	0	0
EU	1151.17	13851	8979.22	25252.85	58462.07	33121.88	34167.08	0	76294.48	107834.17	45295.18	26835.52
NZ	0	0	0	0	0	2401	0	0	0	0	0	0

**060310_+_060210**	2002	2003	2004	2005	2006	2007	2008	2009	2010	2011	2012	2013
Asia	35734	3000	1259	10954	8808	93000	83000	54836	172158	95571	461870.99	161560
Africa	0	76342	0	0	65183	0	1056	87065	193324	60713	0	0
EU	1083487.17	831910.1	561551.22	660013.85	1235485.59	1295835.75	1043363.89	411452.97	186857.48	226364.92	257977.14	212221.85
NZ	0	0	0	0	0	2401	0	0	0	0	0	0

Where does Brazil import Foliage,branches, for bouquets, etc. - fresh from 2002-2013

Foliage,branches, for bouquets	2002	2003	2004	2005	2006	2007	2008	2009	2010	2011	2012	2013
hs92_product_id: 060491	ISR	ZAF	CHN	ISR	ISR	ISR	CHN	ISR	EGY	EGY	EGY	COL
Brazil Import from:	3000	8572	1620	9000	1000	8000	18059	5000	48857	105647	29385	1601
	DEU	ISR			ITA	USA	USA	AUS	USA	ISR		
	83000	1000			3033	2781	288000	14121	2910	91000		
	USA				USA				COL	COL		
	4200				3125				3714.52	2846		
									PER			
									2719			

hs92_product_id: 060491	2002	2003	2004	2005	2006	2007	2008	2009	2010	2011	2012	2013
Asia	3000	1000	1620	9000	1000	8000	18059	5000		91000		0
Africa		8572							48857	105647	29385	
EU	83000				3033							
Australia								14121				

**0602+06031+_060491**	2002	2003	2004	2005	2006	2007	2008	2009	2010	2011	2012	2013
Asia	201490.76	51988.6	166997.04	494861.95	187415.52	516830.47	620200.61	814611.23	1208586.93	1895839.84	3750531.91	3923833.03
Africa	16598.36	167939.87	0	0	65183	21341	4389.02	174713.92	455382	400127.33	98352.2	112341.23
EU	4312749.54	2824503.1	3054245.98	1913142.87	2960179.35	3484936.22	4577248.74	4650901.82	5739291.89	8657890.69	9113665.79	10223232.01
Australasia	0	8186	24464.55	20715.55	3172.11	2401	5833.29	14121	0	0	0	0

Suppl. Table 4: Brazil importation of agricultural and horticultural commodities (in financial volume of $USD ,000) between 2002 to 2013 as represented by the Harmonized System (HS) code 07 for Edible Vegetables and certain roots and tubers (including hs92_product_ID: 070390, 070519, 070810, 070960, 070970, and 070990) from global destinations. International Organisation for Standardisation (ISO) three-letters country codes are used. Countries are grouped by continents (Africa = yellow; Asia = red; Europe = purple; North America = blue; South America = green; Australasia = orange). 'nes' = not elsewhere specified.

Where does Brazil import Leeks & other alliaceous vegetables, fresh or chilled from 2002-2013

Leeks & other alliaceous vegetables, fresh or chilled	2002	2003	2004	2005	2006	2007	2008	2009	2010	2011	2012	2013
hs92_product_id: 070390	CHN	No data	HKG	No data	CHN	No data	No data	CHN	No data	No data	CHN	EGY
Brazil Import from:	208618.28		6563.58		311376.44			16879.12			5040.22	44968
	ESP										THA	CHN
	15714.71										1073	287085.26
	FRA										USA	FRA
	1815.65										4020.14	4700.78
	CHL											
	5989.44											

Where does Brazil import Lettuce, fresh or chilled except cabbage lettuce from 2002-2013

Lettuce, fresh or chilled except cabbage lettuce	2002	2003	2004	2005	2006	2007	2008	2009	2010	2011	2012	2013
hs92_product_id: 070519	AUS	No data	No data	No data	No data	USA	No data	No data	No data	No data	No data	No data
Brazil Import from:	8274					2201.68						

Where does Brazil import Peas, shelled or unshelled, fresh or chilled from 2002-2013

Peas, shelled or unshelled, Fresh or chilled	2002	2003	2004	2005	2006	2007	2008	2009	2010	2011	2012	2013
hs92_product_id: 070810	CAN	KEN	KEN	CAN	CAN	ARG	CAN	CAN	No data	No data	No data	EGY
Brazil Import from:	288958.06	8501	8679	150446.07	141336.83	8100	102782	6323				2110
	USA	CAN	TZA									CAN
	67019.24	15529.19	2579									645509
		URY	CAN									USA
		9351.56	69859.73									74916
			USA									
			10484.61									

Where does Brazil import Peppers (Capsicum, Pimenta) fresh or chilled from 2002-2013

Peppers (Capsicum, Pimenta) fresh or chilled	2002	2003	2004	2005	2006	2007	2008	2009	2010	2011	2012	2013
hs92_product_id: 070960	GBR	CHL	ARG	ARG	URY	No data	No data	GBR	GBR	USA	USA	GMB
Brazil Import from:	4647	7000	11382	9984	19981.93			11639	4406	1322.35	7746.17	2186
			URY	URY				USA	ARG	URY	URY	USA
			18571	7206.67				12500	33620	73790.52	12217.14	9657.08

Where does Brazil import Spinach, Fresh or Chilled from 2002-2013

Plants, live (including their roots),nes	2002	2003	2004	2005	2006	2007	2008	2009	2010	2011	2012	2013
hs92_product_id: 070970	No data	No data	No data	No data	SYR	FRA	No data	No data	No data	No data	No data	DNK
Brazil Import from:					2380	11000						16400

Where does Brazil import Vegetables, fresh or chilled nes from 2002-2013

Vegetables, fresh or chilled nes	2002	2003	2004	2005	2006	2007	2008	2009	2010	2011	2012	2013
hs92_product_id: 070990	FRA	DEU	PRT	KEN	KEN	KEN	EGY	EGY	EGY	EGY	EGY	EGY
Brazil Import from:	16994	5000	28817	9060	20575	32858	53815	10004.8	688345	132617	264416	32158.43
	PRT	ESP	URY	SEN	SEN	LBN	KEN	ITA	FRA	MOZ	CHN	CHN
	14228	7028	70908.65	5500	2623	3292.97	4384	5944	9279	149000	2836.02	54699.88
	USA			IND	GBR	THA	TUR		ITA	IND	DEU	ITA
	3036.49			1742	3585	2504	33498.52		4745.26	14762	1473	2719
				ESP	PRT	ITA	FRA		NDL	FRA	GBR	NLD
				31665	24527	9729.98	1199.05		21221	18087	10445	5898
				GRC	ARG	ARG	ITA		PRT	GBR	NLD	PRT
				10055	32961	1584	3605.02		33975	2934	1789	21697
				ARG	URY		PER		USA	ITA	PRT	ARG
				1825	4290		23020		19469	3515.41	16748	22193.37
										PRT	ARG	
										11482	34619	
										USA	URY	
										7317	10775	
										CHL		
										2904.92		

070390+070519+070810+070960 +070970+070990	2002	2003	2004	2005	2006	2007	2008	2009	2010	2011	2012	2013
Asia	208618.28	0	6563.58	1742	313756.44	5796.97	33498.52	16879.12	0	14762	8949.24	386753.14
Africa	0	8501	11258	14560	23198	32858	58199	10004.8	688345	281617	264416	36454.43
EU	39171.36	12028	0	41720	28112	20729.98	4804.07	17583	39651.26	24536.41	13707	29717.78
Australasia	8274	0	0	0	0	0	0	0	0	0	0	0

Suppl. Table 5: Brazil importation of agricultural and horticultural commodities (in financial volume of $USD ,000) between 2002 to 2013 as represented by the Harmonized System (HS) code 08 for Edible Fruits and Nuts, Peel of Citrus/Melons (including hs92_product_ID: 0804, 081010, and 081090) from global destinations. International Organisation for Standardisation (ISO) three-letters country codes are used. Countries are grouped by continents (Africa = yellow; Asia = red; Europe = purple; North America = blue; South America = green; Australasia = orange). 'nes' = not elsewhere specified.

Where does Brazil import Tropical Fruits from? (Year)

Tropical Fruits	2002	2003	2004	2005	2006	2007	2008	2009	2010	2011	2012	2013
hs92_product_id: 0804	DZA	TUN	TUN	EGY	EGY	TUN	TUN	TUN	TUN	DZA	SEN	BFA
Brazil Import from:	4009.23	275251.76	276424.68	2212.3	105632	957021.9	833056.12	903884.69	1202423.62	13349.8	1851	44744
	TUN	IRN	IND	TUN	SEN	ARE	ARE	IND	IRN	TUN	TUN	TUN
	182876.66	2821.18	3544	353624.4	2554	3036	1045	3977	1293.51	1689404.86	1772142.85	1135394.39
	IRN	LBN	IRN	ARE	TUN	IRN	IRN	IRN	ISR	ISR	ARE	CHN
	16819.43	3153.63	4595.99	30489	354314.97	62754.45	46346.2	4197.68	78713.2	182506.92	1572.46	4484
	LBN	TUR	TUR	IND	IND	LBN	ISR	ISR	LBN	LBN	IRN	IND
	1580.55	463673.09	597406.15	6167	5837	13453.75	44382.37	177319.85	2682.83	6016.58	2913.15	53681
	TUR	ESP	ESP	IRN	IRN	SAU	LBN	LBN	THA	PSE	ISR	IRN
	545114.52	87819.94	124421.98	7754.38	21951.85	15208	2864.66	2661	116664.87	62674.34	187000	14516.18
	ESP	CRI	CRI	LBN	OMN	SYR	THA	THA	TUR	THA	PSE	ISR
	98895.64	242397	145184	1119.84	5540	1934.62	51545.72	50537.47	2052327.6	42834.06	69874.25	86000
	ITA	MEX	CHL	THA	SYR	THA	TUR	TUR	ESP	TUR	THA	LBN
	4021	19890	8779.03	6429.82	1142.7	2736.81	1811880.87	2280247.66	44582.68	2651038.48	113834.85	4920.78
	CRI	USA	COL	TUR	THA	TUR	DEU	ESP	DOM	CRI	TUR	PHL
	1212731	10437.66	1283	919663.15	12978.3	1781910.6	3000	67542.68	6345	18672	2037347.17	22838
	ARG	CHL	ECU	ESP	TUR	ESP	USA	CRI	USA	USA	DEU	THA
	1500	4016.77	6919	113683.99	1062338.67	88543.8	16123.37	3600	100708.94	377064.33	7273	72089.37
	COL	COL		CRI	ESP	USA	CHL	DOM	ARG	CHL	PRT	TUR
	7365	3437		28200	158549.7	7895.82	27430.03	31272	3000	14155	12328.77	1788287.91
				MEX	MEX	ARG		USA	CHL	COL	USA	DEU
				28900	29800	50550		46116.34	8159.28	14749	89221.02	9984
				CHL	USA	CHL		CHL	COL		CHL	ESP
				8228.14	7607.48	29814		25267	12845		45491	51734.31
				COL	ARG			COL	PER		COL	IRL
				2859	22450			1816	4950.5		7862	1639.52
					CHL			PER				USA
					30380			2362				45986.1
					COL							BOL
					2604							22214
					ECU							CHL
					9558							51220.83
												COL
												15531

0804	2002	2003	2004	2005	2006	2007	2008	2009	2010	2011	2012	2013
Asia	563514.5	469647.9	605546.14	971623.19	1109788.52	1881034.23	1958064.82	2518940.66	2251682.01	2945070.38	2412541.88	2046817.24
Africa	186885.89	275251.76	276424.68	355836.7	462500.97	957021.9	833056.12	903884.69	1202423.62	1702754.66	1773993.85	1180138.39
EU	102916.64	87819.94	124421.98	113683.99	158549.7	88543.8	3000	67542.68	44582.68	0	7273	63357.83
Australasia	0	0	0	0	0	0	0	0	0	0	0	0

Where does Brazil import Strawberries, Fresh from?

Strawberries, Fresh	2002	2003	2004	2005	2006	2007	2008	2009	2010	2011	2012	2013
hs92_product_id: 081010	USA		USA	ARG	No data	USA	ETH	USA	CHN	USA	USA	MEX
Brazil Import from:	6333.03		1334.95	17193		15478.54	2540	49111.35	99962	820996.5	719555.8	1417
	CHL						USA	CHL	USA	CHL		USA
	3499.58						63946.83	1098.45	120384	93579.27		644737.36
									CHL			
									116661.53			

Where does Brazil import Fruits, fresh nes from? (Year)

FruitFresh_nes	2002	2003	2004	2005	2006	2007	2008	2009	2010	2011	2012	2013
	ESP	KEN	KEN	ESP	IND	ESP	ESP	ESP	ESP	ESP	DEU	CHN
hs92_product_id: 081090	47291.92	1576	2130	230731.98	8743	167071.6	169597.25	354621.1	590967.97	1243965.08	1731	2066.01
Brazil Import from:	ITA	ESP	IND	ITA	ESP	FRA	FRA	FRA	FRA	FRA	ESP	DEU
	2354740.52	83773.09	2688	3758403.29	182444.89	49661.2	60583.34	15283	111038.14	30829.84	1161665.81	1307
	ARG	ITA	ESP	PRT	FRA	ITA	ITA	ITA	ITA	ITA	FRA	ESP
	29592.2	1671676.76	137469.25	56287	90307.41	3712313.32	4615244.79	6155471.44	10445542.2	15555640.2	24868.94	1786926.23
	CHL	CHL	FRA	USA	ITA	PRT	USA	USA	PRT	PRT	ITA	FRA
	2848317.09	1913314.03	33505.56	19690	3419270.88	8503	126122.19	242005.51	25313	31245.84	10733039.09	21710.39
	COL	COL	ITA	ARG	PRT	USA	NZL	NZL	USA	USA	PRT	ITA
	49214.87	45553.91	1811543.58	32824	22750	63503.48	784517.87	644399.77	390738.13	835523.07	6592.49	16805758.46
	URY		NLD	CHL	ARG	NZL	ARG	CHL	NZL	NZL	USA	PRT
	16960		26126	2897521.95	16002.09	290454.31	444775	8878875.13	1411792.05	2635133.72	1212717.46	21911.05
			ARG	COL	CHL	CHL	CHL	COL	ARG	ARG	NZL	USA
			18726	56877.05	3196742.18	5113206.89	4638548.05	213967.72	20196	33600	2783568.41	873023.11
			CHL		COL	COL	COL		CHL	CHL	CHL	NZL
			2549885.56		55273.18	86715.62	170857.96		10087532.4	12919521.94	16117899.89	2204318.37
			COL		URY				COL	COL	COL	CHL
			35362.04		35406				445562.35	845086.68	901272.81	18144166.58
												COL
												1068701.66

FruitFresh_nes

hs92_product_id: 081090	2002	2003	2004	2005	2006	2007	2008	2009	2010	2011	2012	2013
Asia	0	0	2688	0	8743	0	0	0	0	0	0	2066.01
Africa	0	1576	2130	0	0	0	0	0	0	0	0	0
EU	2402032.44	1755449.85	2008644.39	4045422.27	3714773.18	3937549.12	4845425.38	6525375.54	11172861.3	16861680.96	11927897.33	18637613.13
NZ	0	0	0	0	0	290454.31	784517.87	644399.77	1411792.05	2635133.72	2783568.41	2204318.37

0804+081010+081090

	2002	2003	2004	2005	2006	2007	2008	2009	2010	2011	2012	2013
Asia	563514.5	469647.9	608234.14	971623.19	1118531.52	1881034.23	1958064.82	2518940.66	2351644.01	2945070.38	2412541.88	2048883.25
Africa	186885.89	276827.76	278554.68	355836.7	462500.97	957021.9	835596.12	903884.69	1202423.62	1702754.66	1773993.85	1180138.39
EU	2504949.08	1843269.79	2133066.37	4159106.26	3873322.88	4026092.92	4848425.38	6592918.22	11217444	16861680.96	11935170.33	18700970.96
Australasia	0	0	0	0	0	290454.31	784517.87	644399.77	1411792.05	2635133.72	2783568.41	2204318.37
